# Box–Behnken Design Optimization of Green Extraction from Tomato Aerial Parts and Axillary Shoots for Enhanced Recovery of Rutin and Complementary Bioactive Compounds

**DOI:** 10.3390/antiox14091062

**Published:** 2025-08-29

**Authors:** Simona Marcu Spinu, Mihaela Dragoi Cudalbeanu, Nikola Major, Smiljana Goreta Ban, Igor Palčić, Alina Ortan, Petronela Mihaela Rosu, Narcisa Elena Babeanu

**Affiliations:** 1 Faculty of Land Reclamation and Environmental Engineering, University of Agronomic Sciences and Veterinary Medicine of Bucharest, 59 Marasti Boulevard, 011464 Bucharest, Romania; simona.spinu@fifim.ro (S.M.S.); alina.ortan@fifim.ro (A.O.); 2 Institute of Agriculture and Tourism, 52440 Poreč, Croatia; nikola@iptpo.hr (N.M.); smilja@iptpo.hr (S.G.B.); palcic@iptpo.hr (I.P.); 3 Faculty of Veterinary Medicine, University of Agronomic Sciences and Veterinary Medicine of Bucharest, 59 Marasti Boulevard, District 1, 011464 Bucharest, Romania; 4 Faculty of Biotechnologies, University of Agronomic Sciences and Veterinary Medicine of Bucharest, 59 Marasti Boulevard, 011464 Bucharest, Romania; narcisa.babeanu@biotehnologii.usamv.ro

**Keywords:** tomato waste, rutin, axillary shoots, tomato aerial parts, green extraction, Box–Behnken design (BBD), phytochemical profiling, antioxidant activity

## Abstract

Tomato aerial parts and axillary shoots represent underutilized agricultural residues with promising phytochemical potential. Despite the recognized antioxidant capacity of rutin, current literature lacks optimized, comparative studies on its extraction from distinct tomato vegetative components. This study aimed to maximize the recovery of rutin and other bioactive compounds from tomato plant biomass using green extraction techniques—microwave-assisted extraction (MAE) and ultrasound-assisted extraction (UAE)—optimized through Box–Behnken design (BBD) and Response Surface Methodology (RSM). The extraction process was optimized for three key variables: temperature, solvent concentration, and plant-to-solvent ratio. Four main responses were evaluated: total phenolic content (TPC), total flavonoid content (TFC), antioxidant activity (DPPH), and rutin concentration. The highest rutin content (8614.23 mg/kg) was obtained in extracts from axillary shoots using MAE. Overall, MAE proved more efficient in recovering both primary and secondary metabolites from axillary shoots, while UAE favored the extraction of certain micronutrients and specific amino acids. Cascade extraction further improved the recovery of key compounds such as vitamin E and quinic acid. The comparative profiling of extracts revealed significant phytochemical differences between tomato aerial parts and axillary shoots, addressing a gap in the literature and underscoring the importance of optimized extraction strategies. These findings highlight tomato plant waste as a valuable source of antioxidant compounds and set the stage for future investigations into their biological activities.

## 1. Introduction

Tomatoes (*Solanum lycopersicum* L.) are among the most cultivated vegetables worldwide, thus gaining their place over time among the important vegetable crops. Widely consumed both raw and in processed forms, they currently rank as the second most produced fruit and vegetable crop globally, after apples. Through industrial processing, tomatoes are transformed into a wide range of value-added food products, such as paste, purée, juice, soup, dehydrated tomatoes, and pickled forms.

According to the available statistics, in the last 10 years, the average production of tomatoes for fresh consumption in Romania has varied greatly, taking into account the climatic, economic, and social changes that have occurred. Thus, between 2015 and 2021, there were fluctuations in average production ranging between 469,000 and 500,000 tons, after which there was a significant decrease starting in 2022. Following that, in 2024, it reached an average of 231,000 tons of tomatoes intended for fresh consumption, accentuating a decreasing trend in recent years [1]. At the European level, the area harvested for tomatoes recorded a slight increase compared to the previous year, reaching 395,497 ha in 2023, while in Romania, the same trend of fluctuations was recorded, reaching 17,540 ha [2]. Despite the large-scale production of tomatoes, precise statistics on biomass waste from cultivation and processing are lacking, and only general estimates exist. Current efforts therefore focus on sustainable strategies for valorizing tomato plant residues in line with circular bioeconomy principles.

Tomato biomass includes various plant components such as branches, leaves, stems, bunches, roots, and axillary shoots (“suckers”). This study focuses specifically on the aerial parts of tomato plants, defined here as a mixture of leaves, stems, bunches, and branches, as well as axillary shoots, typically considered post-harvest and pruning residues or waste. In the context of the circular bioeconomy, these plant residues represent an underutilized resource with significant potential for valorization.

Several studies have shown that tomato aerial biomass can be valorized through composting [3], anaerobic digestion for biofuel [4,5], or pyrolysis for biochar production [6,7], the latter being useful in environmental remediation and soil management. More recently, the scientific literature has highlighted innovative approaches to tomato biomass utilization, such as the extraction of bioactive compounds to be used in nutraceuticals [8,9]; development of bioinsecticides [10]; use as a raw material for biodegradable packaging [11]; and even paper production [12] or as a potential source of fermentable sugars [13].

These emerging applications of tomato biomass are linked to its rich composition in primary and secondary metabolites, essential for cellular functions and plant defense [14]. Tomato aerial biomass is particularly abundant in secondary metabolites such as phenolic acids and flavonoids, including p-coumaroyl-caffeoylquinic acid, quercetin-3-O-rutinoside, quercetin derivatives, chlorogenic acid, and neochlorogenic acid. Among the various methods for recovering bioactive compounds, green extraction techniques such as ultrasound-assisted extraction (UAE) and microwave-assisted extraction (MAE) are increasingly favored for their efficiency and sustainability, with UAE promoting cell disruption through cavitation and MAE enhancing mass transfer by rapid heating [15,16].

Añibarro-Ortega et al. [17] reported that rutin is the most abundant flavonoid in the aerial parts of tomato leaves and stems belonging to different accessions of *Solanum lycopersicum* L. (BPGV 12260, BPGV 12437, BPGV 12446, BPGV 12465, BPGV 12906, BPGV 13034) obtained from the Portuguese Genebank (BPGV). Therefore, tomato biomass, an abundant agro-industrial waste in Romania and beyond, can be considered an important source of rutin.

Rutin (quercetin-3-*O*-rutinoside) is a flavonoid with diverse bioactivities, including modulation of intestinal microbiota [18,19], antioxidant [20], and anticancer effects [21]. Despite its potential, rutin remains difficult to isolate, requiring optimized extraction strategies. Current approaches increasingly rely on statistical modeling, particularly Response Surface Methodology (RSM) with designs such as Box–Behnken (BBD) and Central Composite (CCD), successfully applied to optimize parameters for phenolic and flavonoid recovery as well as antioxidant activity [22,23]. UAE and MAE have been used effectively in tomato fruits [24] and seed waste [25] to enhance rutin and related compound recovery under optimized BBD conditions. However, limited data are available regarding the recovery of rutin [9]. Moreover, the specialized literature shows limited research on the optimization of rutin extraction from tomato aerial parts, particularly using modern green extraction methods combined with statistical modeling tools. This gap opens new perspectives for future research.

In this context, the present study investigates the extraction of bioactive compounds from the aerial parts and axillary shoots of *Lycopersicon esculentum* biomass, using assisted extraction techniques (MAE and UAE), followed by process optimization through a Box–Behnken design and quantification of rutin content. To our knowledge, this is the first study to apply Box–Behnken design for the optimization of both MAE and UAE techniques targeting the recovery of rutin-rich extracts from tomato aerial biomass, including axillary shoots. This integrated approach aims to generate extracts with a well-defined chemical profile and enhanced biological activities—antioxidant, antibacterial, and antifungal—with the potential to beneficially modulate the intestinal microbiota. These findings open new perspectives for the high-value valorization of tomato plant waste within a sustainable and circular bioeconomy framework.

## 2. Materials and Methods

### 2.1. Plant Material

**Figure 1:**
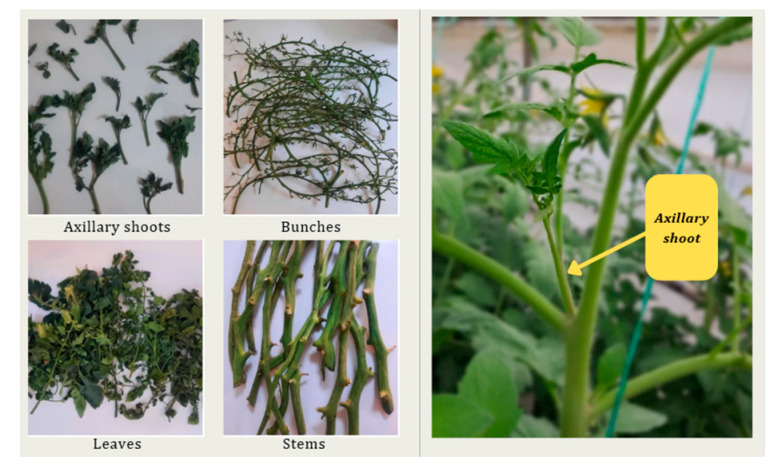
Biomass obtained from aerial parts of tomatoes.

The plant material used in this study was collected from the University of Agronomic Sciences and Veterinary Medicine of Bucharest Research Greenhouse, Bucharest, Romania. It consisted of waste biomass from *Lycopersicon esculentum* (Cheramy RZ F1 hybrid), presented in Figure 1, specifically aerial parts and axillary shoots. The aerial parts contained leaves, stems, and bunches. The axillary shoots resulted from pruning practices, and the aerial parts from tomato crop maintenance.

### 2.2. Plant Material Extraction Methods

For extraction, the fresh plant material was freeze-dried using the Alpha 2-4 LSCplus Christ LyoCube 4-8 Freeze-dryer (Osterode am Harz, Germany), under a 55 h and 30 min program, at a pressure of 0.5 mbar and a temperature of −55 °C. The aerial plant material contained equal quantities of leaves, stems, and bunches. It should be noted that the equal quantities of leaves, stems, and bunches did not represent their natural proportions in a tomato plant but were intentionally standardized in equal parts in order to ensure consistency and comparability of the extraction experiments. After freeze-drying, the plant material was ground using a knife mill at 2000 rpm for t = 10 s for leaves and axillary shoots and t = 20 s for stems and bunches. The humidity of plant material used was below 3%, as measured by the Partner MAC50 moisture analyzer (Bucharest, Romania). Two extraction methods were selected: ultrasound-assisted extraction (UAE) and microwave-assisted extraction (MAE), based on the promising results previously obtained by our research group using other types of plant material. After extraction, the extracts were subjected to vacuum filtration, concentration (using a Microvap 118 Nitrogen Evaporator, Organomation^®^, Berlin, MA, USA), and then freeze-drying until further analysis.

#### 2.2.1. Ultrasound Assisted Extraction (UAE)

Ultrasound-assisted extraction (UAE) was performed using an ultrasonic processor (Sonics Vibra-CellTM VCX 750, SONICS & MATERIALS, INC., Newtown, CT, USA). The optimization process was carried out using the following extraction parameters: a fixed extraction time of 1 h; plant-to-solvent ratios of 1:20, 1:30, and 1:40; temperatures of 40 °C, 55 °C, and 70 °C; and ethanol concentrations of 50%, 75%, and 100%. These parameters were used for both the aerial parts mixture and axillary shoots. The total time was distributed as follows: 3 sequences of 10 min pulses (blocks of 59 s pulses on and 10 s pulses off) alternated with 2 sequences of 15 min rest, at an amplitude of 35% (at 40 °C and 55 °C) and 65% (at 70 °C). The ultrasonic processor operated at 750 W and 20 kHz.

#### 2.2.2. Microwave Assisted Extraction (MAE)

Microwave-assisted extraction (MAE) with a duration of 1 h was performed using Milestone Ethos Easy equipment (Milestone Srl, Bergamo, Italy). Based on the experimental design, the following extraction parameters were chosen for process optimization plant-to-solvent ratio: 1:20, 1:30, and 1:40; temperature: 70 °C, 98 °C, and 125 °C; hydroalcoholic solvent concentrations: 50%, 75%, and 100% ethanol (*v*/*v*). These parameters were used for both the aerial parts mixture and axillary shoots. The microwave power was 550 W.

#### 2.2.3. Cascade Extraction

Cascade extraction was applied to both types of plant material after determining the optimal extraction parameters. In the first variant, ultrasound-assisted extraction (UAE) was followed by microwave-assisted extraction (MAE), applying the plant-to-solvent ratio and solvent concentration optimized parameters identified for MAE; the resulting extracts were designated as C1ap (aerial parts) and C1as (axillary shoots). In the second variant, the same extraction sequence was maintained—UAE followed by MAE—but with extraction times reduced by half, leading to the C2ap and C2as extracts. This approach aimed to evaluate whether shortened extraction durations could still ensure efficient recovery of bioactive compounds under optimized conditions.

### 2.3. BBD Experimental Design

#### 2.3.1. Experimental Design for Optimization of Bioactive Compounds Recovery from Aerial and Axillary Shoots Tomato Waste

The optimization of bioactive compound extraction from tomato plant material was carried out using a Box–Behnken design (BBD) in combination with Response Surface Methodology (RSM). A total of 17 experimental runs were performed, including 5 centroids, three levels, and three independent variables. Solvent concentration (%), solvent-to-plant ratio and temperature (°C) were selected as independent variables. This optimization process was carried out using the Design Expert software package version 13 (Stat-Ease, Inc., Minneapolis, MN, USA). Each experimental run was performed in triplicate. The response variables monitored were the total polyphenol content (Y1—TPC (mg GAE/kg)), the total flavonoid content (Y2—TFC (mg QE/kg)), Y3—antioxidant activity measured by the DPPH method (%) and Y4—rutin content (mg/kg). In total, four separate optimizations were conducted: two for the aerial parts and two for the axillary shoots, corresponding to each extraction method (MAE and UAE). The experimental designs used for each extraction method are presented in Table 1.

#### 2.3.2. Validation of the BBD Model

Using RSM, the results of the model applied for each situation were obtained as 3D surface plots. For each studied case, the significance of the model coefficients and the global model (R^2^, R^2^-adjusted, *p*) was verified by selecting a confidence level of 95%, using ANOVA and summary-of-fit tests. Significant differences were considered for *p* < 0.05.

After optimization, extracts were prepared in triplicate for each studied case, using the parameters corresponding to the identified optimal solution with the highest desirability value, in order to confirm the model predictions.

### 2.4. Determination of the Total Phenolic Content (TPC) and Total Flavonoid Content (TFC)

The determination of the total phenolic content (TPC) and total flavonoid content (TFC) in the extracts from the aerial parts and axillary shoot wastes was carried out by micro-spectrophotometry using FLUOstar^®^ Omega microplate reader (Omega Software package (v. 5.10 R2), BMG LABTECH, Ortenberg, Germany). These methods have previously been applied and validated by our research group in similar studies involving plant-based materials.

The total phenolic content (TPC) of the samples was determined using the Folin–Ciocalteu reagent method. The reaction was performed by mixing the plant extract with 1 N Folin–Ciocalteu reagent and 20% sodium bicarbonate. After incubation for 30 min at room temperature, the absorbance was measured at 760 nm. The TFC was determined by reacting the plant extract with 2% aluminum chloride, followed by a 15 min incubation at room temperature. The absorbance was then recorded at 415 nm. The TPC results were expressed in mgGAE/kg dry weight (dw), and the TFC results in mgQUE/kg dw. For each sample, the measurements were performed in triplicate.

### 2.5. Rutin Content Quantification by UPLC

The identification and quantification of rutin in the studied plant extracts was performed using ultra-high performance liquid chromatography (UPLC) equipment (Waters Acquity UPLC^®^ I Class, Milford, MA, USA). The system is provided with a detector associated with a photodiode array (PDA) and a Zorbax Eclipse Plus C18 column (Agilent Technologies, Santa Clara, CA, USA). The dimensions of the chromatographic column are 4.6 × 150 mm, and the particle size is 5 µm. The two solvents of the mobile phase were water with 0.1% formic acid (solvent A) and acetonitrile with 0.1% formic acid (solvent B). The gradient program started with 90% solvent A and gradually reached 0%, while solvent B varied in the opposite direction. During the analysis, the flow rate was constant (0.8 mL/min) for a total analysis time of 30 min.

### 2.6. Antioxidant Activity Evaluation by DPPH Assay

Using microspectrophotometric techniques, the free radical scavenging capacity of DPPH (1,1-Diphenyl-2-Picrylhydrazyl) was determined to evaluate the antioxidant potential of the extracts obtained from the studied plant waste. Thus, the plant extract was mixed in equal parts with 250 μM DPPH solution and incubated in the dark for 30 min at room temperature. The absorbance was registered at 517 nm.

The results of the measurements, performed in triplicate, were expressed as percentages using the well-established mathematical equation:

DPPH scavenging activity (%) = (Abs_control_ − Abs_sample_)/Abs_control_ × 100 (1)

### 2.7. Identification and Quantification of Primary and Secondary Metabolites from Optimized Extracts

The identification of primary and secondary metabolites in the optimized extracts from aerial parts and tomato axillary shoots was carried out using a Shimadzu LCMS—8045 system. This system includes a controller (Shimadzu SCL-40), a degasser (Shimadzu Nexera DGU-405), two solvent delivery units (Shimadzu Nexera LC-40DX3), an autosampler (Shimadzu Nexera SIL-40CX3), a thermostated column compartment (Shimadzu Nexera CTO-40C), and a QqQ mass spectrometer (Shimadzu LCMS8045).

The polyphenolic analysis of lyophilized extracts was conducted according to Major et al. [26], with slight modifications. Separation was achieved on a C18 core-shell column (2.1 mm × 150 mm, 2.7 μm; Agilent, Palo Alto, CA, USA) maintained at 37 °C. A 0.5 μL sample was injected, and compounds were separated using a linear gradient elution with mobile phase A (water with 0.1% acetic acid) and mobile phase B (methanol with 0.1% acetic acid), at a flow rate of 0.35 mL/min. The gradient program was as follows: 0–0.75 min, 98% A; 0.75–15 min, 98% A to 50% A; 15–15.1 min, 50% A to 0% A; 15.1–20 min, 0% A; 20–20.1 min, 0% A to 98% A; and 20.1–25 min, 98% A.

The primary metabolite profile was analyzed according to Polić Pasković et al. [27], on a Discovery^®^ HS F5-3 column (2.1 mm × 150 mm, 3 μm core–shell; Sigma-Aldrich, St. Louis, MO, USA), maintained at 37 °C. A 1 μL aliquot was injected, and separation was achieved using a linear gradient of mobile phase A (water with 0.1% formic acid) and mobile phase B (acetonitrile with 0.1% formic acid) at a flow rate of 0.25 mL/min. The gradient conditions were as follows: 0–2 min, 100% A; 2–5 min, 100% A to 75% A; 5–11 min, 75% A to 65% A; 11–15 min, 65% A to 5% A; 15–20 min, 5% A; 20–20.1 min, 5% A to 100% A; and 20.1–25 min, 100% A.

Identification and quantification of the targeted metabolites were carried out by comparing retention times, characteristic precursor/product ion transitions, and peak areas with those of authentic standards.

### 2.8. Identification and Quantification of Micro and Macro-Nutrients from Optimized Extracts

The analysis of the elemental composition (Al, B, Ca, Cd, Co, Cr, Cu, Fe, K, Li, Mg, Mn, Mo, Na, Ni, P, S, Se, Si, Zn) of tomato waste extracts was performed using ICP-OES equipment (ICPE 9820, Shimadzu Corporation, Kyoto, Japan). Before analysis, the samples were subjected to microwave-assisted digestion (Ethos UP, Millestone Srl, Milan, Italy). The analysis method adopted for this type of vegetable sample is similar to that previously described by Palčić et al. [28]. Briefly, 200–250 mg of the lyophilized extract was subjected to digestion with 6 mL concentrated HNO_3_ and 2 mL H_2_O_2_ 30%. Subsequently, the digested samples were transferred to 25 mL volumetric flasks and brought to volume with ultrapure water. To assess method accuracy, four certified reference materials from the WEPAL dried plant material program (WEPAL, Wageningen, The Netherlands) were utilized. Element detection was carried out using an ICP-OES equipped with both axial and radial viewing. The instrument was operated under the following conditions: 1.15 kW of an RF power, 12 L/min of a plasma flow rate, 0.5 L/min of an auxiliary gas flow rate, and 0.5 L/min of a nebulizer flow rate. Sample solutions were introduced into the plasma via a concentric nebulizer and a cyclonic spray chamber. High-purity argon (99.999%, Linde Gases, Ananindeua, PA, Brazil) was used for both optics purging and plasma generation.

### 2.9. Statistical Analysis

The obtained results were statistically analyzed using the GraphPad software package (v. 10.3.0). Analysis of variance (ANOVA) and Tukey’s test were employed, both with a significance threshold of 5% (α = 0.05). The obtained results are presented as the mean ± standard deviation (SD).

## 3. Results

### 3.1. BBD Model Optimization

In order to maximize the extraction of bioactive compound content, particularly rutin, the extraction process of aerial parts and axillary shoots of tomato was optimized by RSM, using the Box–Behnken design applied to ultrasound-assisted (UAE) and microwave-assisted (MAE) processes. Table S1 (Supplementary Material) comprises the BBD matrix for each extraction method and plant material studied. The BBD matrix contains the independent and dependent variable values.

As could be observed also in Table S1, during the optimization of the TPC, values ranged from 3.27–7.69 mg GAE/g DW at UAEap, 4.62–14.69 mg GAE/g DW at UAEas, 2.85–6.39 mg GAE/g DW MAEap and 4.10–8.61 mg GAE/g DW at MAEas. The TFC varied from 0.95–2.25 mg QE/g DW at UAEap, 1.14–2.96 mg QE/g DW at UAEas, 0.98–1.94 mg QE/g DW at MAEap, 1.16–3.50 mg QE/g DW at MAEas. As regards DPPH radical scavenging percentage, it ranged from 43.37–98.14% for UAEap, 83.16–91.44% for UAEas, 67.57–90.70% for MAEap, and 86.27–91.78% for MAEas. Furthermore, the quantified rutin content ranged within the following values: 1.85–6.49 mg/g UAEap, 3.16–7.72 mg/g UAEas, 2.96–6.22 mg/g MAEap, and 3.97–8.62 mg/g MAEas.

The experimental data analysis was performed, and in each of the 4 studied situations (UAE for aerial parts and axillary shoots (UAEap and UAEas encoded) and MAE for aerial parts and axillary shoots (MAEap and MAEas encoded) a 2nd order polynomial equation was generated for each investigated response. The final equations in terms of coded variables obtained, 16 in total, are presented in Table S2 (Supplementary Material). For each experimental model assigned to each response and each case studied, the quadratic model with an R^2^ value greater than 0.98 was the best fit, as can be seen in Table 2.

As can be seen in Table 2, the values of R^2^ and predicted R^2^ are close to 1, while the difference between adjusted R^2^ and predicted R^2^ is less than 0.2, indicating that for all the studied situations, the experimental and predicted values are correlated, and the model is fitted. Also, the model fit was evaluated based on the adequate precision values, with a desirable value being greater than 4; in all cases tested, this was above 18 (the lowest value was 18.7854 for DPPH response at MAEas, and the highest was 77.9718, also for DPPH response at UAEas). The coefficient of variation, also shown in Table 2, indicates a good accuracy of the studied models. The low CV values, ranging from 0.16–4.70%, suggest a low variability of the experimental data compared to the predicted ones, which confirms the reliability of each RSM model. In optimization studies, a CV lower than 10% is considered appropriate for robust predictive models. According to the ANOVA results, the developed models were found to be significant for the identified F values, which are associated with *p* values lower than 0.05, in all studied cases. According to the F values presented in Table 2, the differences between the means of the 17 experimental runs are not likely to be random, with only a 0.01% probability that these could be attributed to noise. This confirms the validity of each developed model.

**Figure 2:**
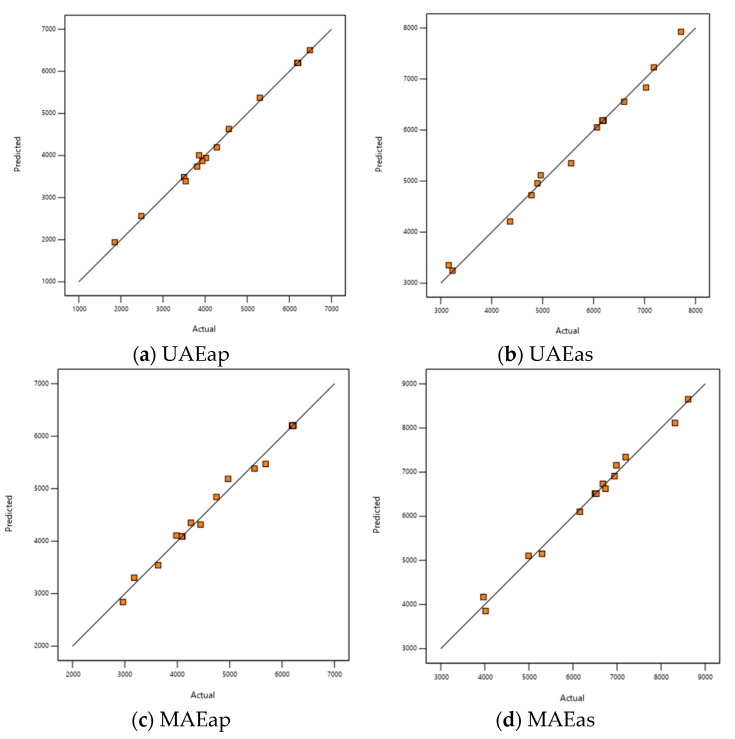
Diagnostic plot of predicted versus experimental values for rutin content response.

The accuracy of the regression model was also evaluated based on the correlation between the predicted and experimental responses. Figure 2 illustrates this comparison for rutin content, separately for each extraction method.

The linear dependence indicates the ideal case where predicted values perfectly match actual (experimental) values. In Figure S1 (Supplementary Material) are presented the diagnostic plots for TPC, TFC, and DPPH responses. The data points are closely clustered around the diagonal line, suggesting a good model fit. This plot helps assess the effectiveness of the developed regression model.

### 3.2. The Influence of the Independent Variables on the Studied Responses

The optimization of extraction parameters is crucial for maximizing the concentration of bioactive compounds. Among these parameters, solvent concentration, extraction temperature, and plant-to-solvent ratio play key roles, as they directly influence both the solubilization capacity and the diffusion rate of the target compound [29]. An insufficient plant-to-solvent ratio may limit the extraction efficiency by restricting mass transfer, whereas an excessively high ratio can result in solvent consumption and dilution effects [30]. Extraction temperature enhances yield by increasing molecular motion and solvent penetration, thus accelerating the diffusion of bioactive compounds from plant matrices [31]. However, sensitive bioactive compounds may suffer thermal degradation if subjected to very high temperatures. Also, the polarity of the compounds of interest is the key factor in selecting the solvent concentration, as it directly influences both solubility and selectivity.

Analyzing the linear terms in each equation of the developed model (see Table S2 Supplementary Material), it could be observed that the water-solvent ratio (%) had a significantly negative influence on rutin content and a significantly positive influence on DPPH antioxidant activity in the case of all four sets of extractions.

Temperature and solvent-to-plant ratio proved to have a significantly positive influence on the rutin content in the case of UAEap, UAEas, and MAEas. Also, TPC was significantly positively influenced by temperature at MAE and UAE of axillary shoots and also at MAE of aerial parts but significantly negatively influenced at UAE of aerial parts. Moreover, this independent parameter significantly positively influenced TFC at UAE of both types of plant material and at MAE of axillary shoots, and significantly negatively influenced TFC at MAE of aerial parts.

The plant-to-solvent ratio had a significant positive effect on TFC across all performed extractions, as well as on the rutin content in MAEas, UAEas, and UAEap.

Overall, the two independent variables, temperature and plant-to-solvent ratio, exerted a greater positive effect on the studied responses compared to the solvent concentration.

**Figure 3:**
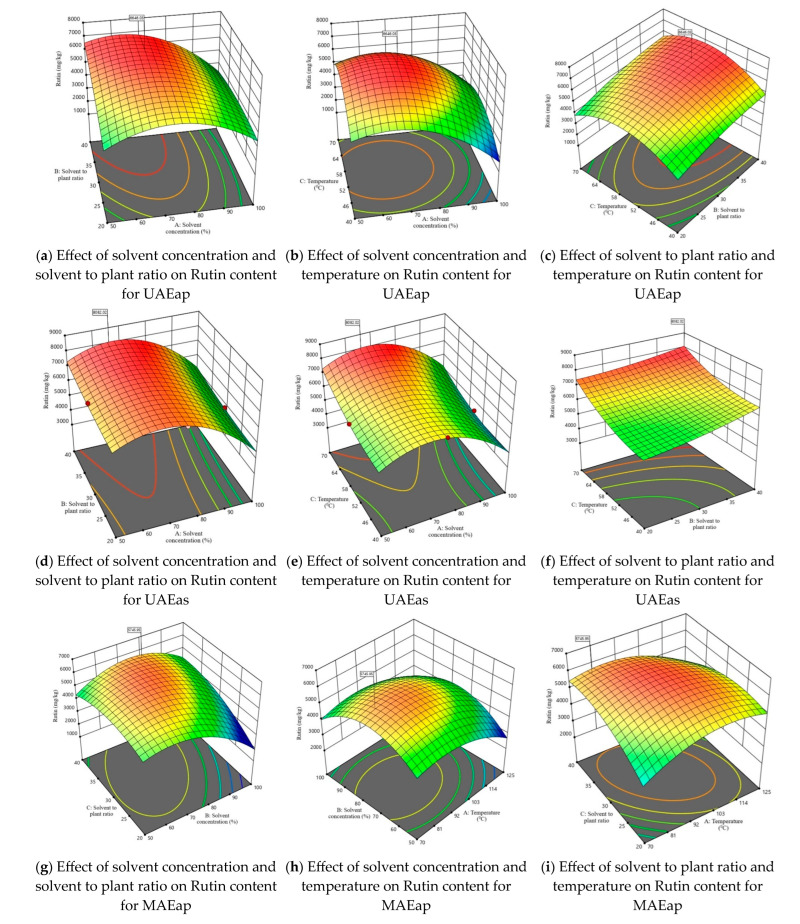

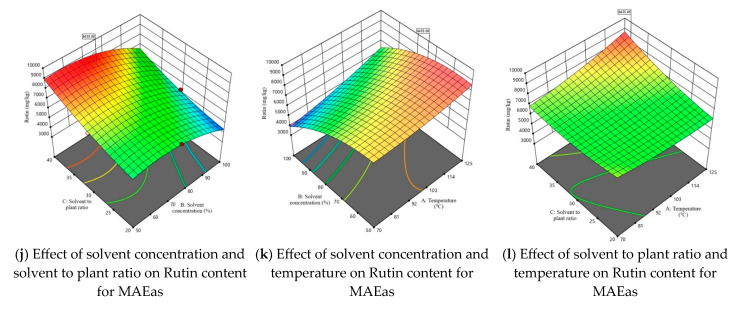
3D response surface plots corresponding to the response regarding optimization of rutin content present in tomato waste (red color—maximum values; green-yellow colors—intermediate values; blue color—minimum values).

Considering that one of the main objectives of this study was to maximize the extraction of rutin from tomato plant waste, Figure 3 illustrates the 3D response surface plots for this compound. The response surfaces for the other three parameters—TPC, TFC, and DPPH—are presented in Figures S2–S4. The graphs in Figure 3 illustrate for both the axillary shoots and the aerial parts mixture, the combined effects of the independent variables, shown two at a time. The maximum predicted values for TPC, TFC, DPPH, and rutin content were identified using the desirability function. This approach allows the determination of optimal conditions by combining the independent variables, thus achieving a balance between them and the dependent variables. The selected optimal solution corresponds to a desirability function as close as possible to 1.

In Table 3 are presented optimum extraction conditions and desirability function values for each extraction method used. Following the experimental design optimization, final ultrasound-assisted and microwave-assisted extractions were performed under the optimized conditions summarized in this table. Given the aim of maximizing all responses simultaneously, an importance level of 5 was given to rutin content, 4 to TFC, 3 to TPC, and 2 to DPPH.

### 3.3. Validation of the Optimized Models

Optimal conditions for both of the selected extraction methods—MAE and UAE—were established through a numerical optimization. Thus, using numerical optimization ramps, the optimal values for extraction temperature, solvent concentration, and solvent-to-plant ratio were determined in order to simultaneously maximize the response variables. To validate the 4 response variables predicted by optimization and presented in Table 3, new extractions of the same plant material were carried out in triplicate for each method, under the optimal conditions identified.

The new set of experimental results (Table 4) obtained for the responses TPC, TFC, DPPH, and rutin content showed values very close to the maximum predicted response values by the selected quadratic model, confirming the reliability and effectiveness of the MAE and UAE extraction methods optimized by BBD-RSM.

By adopting this approach, the number of experiments required for the extraction of bioactive compounds with potential antioxidant properties can be reduced without compromising the validity of the results.

Cascade extraction was performed following model validation, using for both UAE and MAE the previously identified optimal parameters. The specific extraction conditions applied in this stage are summarized in Table 5.

### 3.4. Extracts Characterization Obtained Under Optimized Parameters

#### 3.4.1. Identification and Quantification of Primary Metabolites

In this study, LS-MS analysis was performed using positive ion mode for identification and quantification of the primary metabolites present in the extracts. By performing this chromatographic analysis, it was possible to identify a number of 27 primary metabolites, which are presented in Table 6.

Primary metabolites are directly involved in the regulation of various cellular functions in protein synthesis, respectively, in energy metabolism. In the present study, 15 amino acids with an essential role in protein synthesis and in the processes of adaptation to stress factors were identified, including serine, glycine, glutamine, aspartic acid, alanine, threonine, proline, histidine, lysine, arginine, valine, leucine, isoleucine, phenylalanine, and tryptophan. In particular, proline acts as an osmoprotectant and mitigates reactive oxygen species under stress; phenylalanine feeds the phenylpropanoid pathway (serving as a precursor of rutin); tryptophan is a precursor of indole-3-acetic acid; glutamine and aspartate mediate nitrogen assimilation and shuttling; arginine supports polyamine and nitric oxide biosynthesis; histidine contributes to metal binding; and the branched-chain amino acids (valine, leucine, and isoleucine) can fuel respiration during stress. In addition, organic acids such as citric acid, malic acid, and quinic acid were also identified in the studied extracts, which are known to be key intermediates in energy-generating metabolic pathways. Specifically, citric and malic acids are core tricarboxylic acid (TCA) cycle intermediates linked to energy/redox balance and pH/ion homeostasis, whereas quinic acid is a biosynthetic precursor for chlorogenic acid and other phenylpropanoids. Moreover, the presence of vitamin E in the extracts indicates its potential contribution to antioxidant defense mechanisms in plant tissues such as leaves, stems, bunches, and axillary shoots. Vitamin E was found in higher concentrations in the axillary shoots compared to the aerial parts. The maximum concentration was achieved through full-time cascade extraction (C1as—70.13 mg/g dried extract). In contrast, for the aerial parts, the highest concentration was obtained by MAE (39.95 mg/g dried extract). In addition, the detection of the phytohormone indole-3-acetic acid (UAEas—2.95 mg/g dried extract) supports its known role in regulating plant cell growth and differentiation. Moreover, a nucleobase (cytosine, MAEap—0.19 mg/g), an alkaloid (histamine, MAEap—0.34 mg/g), and a quaternary ammonium compound (choline, UAEas—1.65 mg/g) were identified.

#### 3.4.2. Identification and Quantification of Secondary Metabolites

Liquid chromatography coupled with mass spectrometry was applied to analyze the secondary metabolites present in the studied extracts. In this analysis, positive ionization was used for a better identification of phenolic acids, flavonoids, and other existing compounds, respectively, their derivatives. Thus, it was possible to selectively detect and accurately identify a wide range of bioactive compounds present in the aerial parts of tomato plants, based on retention times and characteristic mass-to-charge ratios (m/z).

The identification and quantification of secondary metabolites from the aerial parts of the tomato plant, namely a mixture of leaves, bunches, branches, and stems, as well as from the axillary shoots, was carried out.

According to the results obtained, the presence of several classes of secondary metabolites can be observed, including phenolic acids, flavonoids, and lignans, with concentrations differentiated by the plant component studied (mixture of aerial parts or axial shoots). From the flavonoid class, flavonols, anthocyanins, and flavones were present in the extracts.

A total of 25 secondary metabolites were identified (Table 7), with concentrations ranging between 0.002 and 21.120 mg/g of lyophilized extract. The lower limit was attributed to baicalin, and the upper to rutin. Analyzing the identified compounds, it can be stated that the phytochemical composition is dominated by 11 phenolic acids and 9 flavonols, representing together almost the entire content of secondary metabolites.

#### 3.4.3. Mineral Profile of Extracts

The mineral content of tomato aerial parts and axillary shoot extracts was determined using ICP-OES, providing insights into the elemental composition of the analyzed samples. This method allows the simultaneous analysis of several essential and potentially toxic elements, providing relevant information about the mineral composition of plant samples. Argon plasma generates the excitation of atoms and ions in the sample, the resulting optical emissions being detected at wavelengths specific to each element. High sensitivity, simultaneous analysis of multiple micro- and macroelements, or a wide detective range are just some of the advantages of this technique.

A total of 16 minerals (Table 8) were identified in the aerial parts and axillary shoots of tomato plant. Among these 16 minerals, six macronutrients (Ca, K, Mg, Na, P, and S), seven micronutrients (B, Cu, Fe, Mn, Mo, Zn, and Se) and three beneficial elements (Al, Li, and Si) are observed.

Among macronutrients, the most abundant was potassium (77.98 ± 3.73 g/kg dry extract), followed by phosphorus (7.37 ± 0.05 g/kg dry extract) and sulfur (3.39 ± 0.06 g/kg dry extract). In the extracts from the mixture of aerial parts, K was detected in higher concentrations than in the extracts obtained from axial shoots. Also, P and S presented higher concentrations in the case of extracts obtained from axial shoots. Comparing the two extraction methods, UAE was more efficient in the recovery of macronutrients.

Considering micronutrients, zinc was the major element (105.39 ± 2.90 mg/kg dry extract), followed by boron (56.29 ± 0.40 mg/kg dry extract) and copper (22.29 ± 0.37 mg/kg dry extract). Similarly, UAE demonstrated greater efficiency in the extraction of micronutrients. B and Cu were predominantly found in the aerial parts, whereas Zn was identified in higher concentrations in the axillary shoots.

Among the beneficial elements, Al and Li were found in higher concentrations and may contribute to various ecological functions. However, Si (60.24 ± 2.23 mg/kg dry extract) was predominantly detected in the extract obtained through the C2ap method. Zn, Cu, and B were detected in nutritionally relevant concentrations [32,33,34]. Potassium was the most abundant macronutrient, but its values did not fall into the optimal nutritional ranges [33,34]. However, the high values in the case of extracts obtained by UAE prove that this method is more suitable for the valorization of potassium from the studied plant waste. These findings support the potential use of this biomass as a complementary source of mineral elements for further applications.

## 4. Discussion

Using an experimental design and response surface methodology (RSM), we optimized UAE and MAE for extracting bioactive compounds from tomato aerial parts and axillary shoots, with emphasis on rutin. Beyond identifying optimal parameters, these data highlight matrix-specific advantages of each technique: MAE of axillary shoots yielded the highest rutin, whereas UAE of the mixed aerial parts showed the lowest; axillary-shoot extracts obtained by MAE also generally exhibited higher TPC, TFC, and DPPH inhibition. These trends suggest that the choice of extraction should be target driven, reflecting differences in tissue composition and mass-transfer mechanisms.

These findings underscore the effectiveness of MAE for valorizing tomato biomass by enhancing recovery of antioxidant compounds. Mechanistically, MAE provides rapid, uniform heating of solvent and matrix with precise control of power and agitation, accelerating rutin diffusion while limiting thermal degradation [31,35]. Consistent with this, prior studies reported higher rutin yields with microwave-based extraction than with conventional methods in cassava leaves and in *Ribes mandshuricum* (Maxim.) Kom. leaves [36,37].

When employing specialized microwave extraction systems similar to the equipment used in this study, several reports have revealed significantly elevated flavonoid content from various plant materials, such as *Oroxylum indicum* (L.) Kurtz leaves [38], black chokeberry fruit [39], monofloral honeys [40], or grape skins and seeds [41].

Comparing the optimal conditions presented in Table 3 with those presented in the literature, UAE determines high concentrations of TPC and TFC, as demonstrated by Vo et al. [42], who identified optimal extraction from passion fruit peels at a plant-to-solvent ratio of 1:30–1:40 and a temperature of 60 °C. In our study, regarding both plant materials, optimal conditions (plant-to-solvent ratio approximately 1:36–1:40 and temperature about 56–70 °C) for UAE led to high concentrations of TPC (UAEap—7.68 ± 2.23 mg GAE/g, respectively, UAEas—12.27 ± 11.54 mg GAE/g) and TFC (UAEap—2.26 ± 1.87 mg QE/g, respectively, UAEas—3.06 ± 2.15 mg QE/g).

Additionally, El Adnany et al. [43] confirmed that a 60% ethanol concentration is ideal for obtaining significantly elevated levels of TPC, TFC, and antioxidant activity measured by DPPH assay. In contrast, for MAE, recent studies have indicated that a 75% ethanol solvent is optimal for maximizing TPC, DPPH, and TFC values, as shown by Febriani et al. [44].

Moreover, according to data reported in the literature and supported by our previous findings, it remains challenging to draw a clear boundary between MAE and UAE. Each technique presents advantages and limitations, and in many cases, the differences in extraction efficiency are not statistically significant. These outcomes largely depend on the type of plant material, its matrix characteristics, and the cultivation conditions.

As shown in Table 6, the primary-metabolite profile underscores the biochemical complexity of tomato aerial parts and axillary shoots. LC-MS identified 15 amino acids, but organic acids predominated, with quinic acid most abundant, followed by malic acid, together with vitamin E. In axillary shoots, cascade extraction yielded the highest levels of these constituents, consistent with enhanced cellular protection, particularly against oxidative stress. The central role of the organic acids in energy-generating pathways and the presence of vitamin E support the maintenance of physiological balance and adaptive responses, reinforcing the valorization potential of this biomass as a source of nutritionally and functionally relevant compounds.

It is observed that, in the case of extracts obtained in cascade from the axillary shoots, the concentrations of these compounds are the highest, supporting their potential contribution to cellular protection mechanisms, particularly against oxidative stress. This distribution indicates that organic acids are present in higher quantities compared to amino acids, highlighting their active involvement in the physiological processes of defense and adaptation of plants.

Comparing the extraction method used for the three identified organic acids, malic acid was identified in the aerial parts of tomatoes at the highest concentration (58.93 mg/g dried extract) using MAE and at the lowest (48.93 mg/g dried extract) using UAE. However, these differences were not statistically significant between MAEap, C2ap, and C1ap.

Similarly, in the case of axillary shoots, the highest malic acid content (55.75 mg/g dried extract) was also obtained using MAE, and the lowest with cascade extraction (C2as). Nevertheless, no statistically significant differences were observed between MAEas, UAEas, and C1as.

On the contrary, the highest concentrations were observed for quinic acid in axillary shoots, where cascade extraction methods proved significantly more effective than MAE and UAE. Notably, quinic acid was found in the aerial parts solely after MAE, suggesting that this technique may enhance its release from the plant matrix.

For the extracts obtained from aerial parts, MAE favored the extraction of cytosine, showing statistically significant variances compared to the other extraction methods, while the cascade extraction (C2ap) proved advantageous for sarcosine.

In extracts obtained from axillary shoots, UAE demonstrated favorability for 2-aminobutyric acid, indole-3-acetic acid, histamine, choline, isoleucine, phenylalanine, and tryptophan. Cascade extraction yielded favorable outcomes for serine, threonine, and valine as observed in the C2as sample. These findings are consistent with previous studies, which also reported the presence of amino acids, particularly in tomato leaves [8,45]. The occurrence of these compounds may result from protein degradation or from endogenous biosynthetic pathways, as suggested by the existing literature on tomato leaf composition. The high amino acid content identified in our samples may be attributed to the fact that tomato leaves are metabolically active tissues, rich in proteins and characterized by intense amino acid metabolism, further supporting the observed metabolite profile [46,47]. To the best of our knowledge, no studies have been identified in the reviewed literature that directly compare the content of primary metabolites between extracts obtained from tomato aerial parts and those from axillary shoots. This gap highlights a novel perspective for the efficient and sustainable valorization of this underutilized plant material, often considered agricultural waste.

According to the results obtained regarding the analysis of secondary metabolites, esculin was identified only in the aerial parts, missing in the extracts from axillary shoots. Esculetin, caffeic acid, and ferulic acid were identified in the aerial parts mixture exclusively when applying the MAE extraction, suggesting that this method may be particularly efficient for the recovery of hydroxycinnamic acids and coumarins. In contrast, kaempferol-7-O-glucoside and kaempferol-3-*O*-glucoside were detected exclusively in axillary shoots, suggesting that these plant parts are a unique source of these flavonol glycosides. However, this distribution may also vary depending on the tomato variety. UAE proved effective for the recovery of spiraeoside, which was not detected in the MAE-derived extract. This finding underscores the complementary nature of these extraction techniques in recovering specific compounds.

Rutin was the predominant secondary metabolite identified, with the highest concentration recorded in the cascade extracts from aerial parts with halved extraction time, C2ap (21.12 ± 0.77 mg/g dried extract). Malvidin was the second most abundant compound, reaching 18.95 ± 0.26 mg/g dried extract under the same conditions. Notably, the third most abundant compound, chlorogenic acid, was detected in higher concentrations in the axillary shoots’ extracts obtained by UAE (13.85 ± 0.25 mg/g dried extract), suggesting that UAE is particularly efficient in recovering certain anthocyanins or related flavonoids from axillary shoots’ tissues.

Solaberrieta et al. [25] reported that, for tomato seeds, ethanolic MAE was more efficient than UAE for chlorogenic acid and rutin. In our study, the mixed aerial parts showed the same trend for chlorogenic acid (MAE > UAE), whereas in axillary shoots, UAE was more efficient. For rutin, MAE gave higher values in the aerial parts, while in axillary shoots there was no significant difference between MAE and UAE. The cascade extraction gave the highest overall rutin, exceeding single-step MAE and UAE, with a time effect: full-time cascade favored axillary shoots, whereas half-time cascade favored the aerial parts. These differences may be related to variety, extraction conditions, and growing environment.

Similarly, the study conducted by Wawoczny et al. [48] confirmed the abundance of rutin as the main secondary metabolite in tomato leaves, using deep eutectic solvents for extraction. Their findings are comparable to those obtained in the present study, further validating the effectiveness of the extraction methods employed. In the study conducted by Röhlen-Schmittgen et al. [49], the application of UAE to tomato leaves confirmed the presence of rutin in the obtained extracts, further supporting the potential of green extraction methods for the recovery of this compound.

Considering all the above, it can be concluded that both the plant component used for extraction and the extraction technique significantly influence the phytochemical profile, in terms of both quality and quantity. This highlights the importance of optimizing the extraction process to maximize the yield of target compounds.

Secondary metabolites play an essential role in biological processes, as demonstrated by numerous studies. For example, Kim et al. [50] investigated the antimicrobial activity of phenolic acids extracted from tomato leaves against the pathogens *Fusarium oxysporum* f. sp. *lycopersici*, *Glomerella cingulata,* and *Rhizoctonia solani.*

The high rutin content identified in both tomato aerial parts and axillary shoots supports their potential as sustainable plant-based sources of this multifunctional flavonoid. The findings align with previous studies reporting the broad spectrum of biological activities attributed to rutin, including antidiabetic [51,52], cardioprotective [53,54], anti-obesity [18,55], anti-inflammatory [56], anticancer [57], and gut microbiota-modulating effects [58,59]. In light of these results, the tomato plant biomass analyzed in this study proves to be a promising and sustainable source of bioactive compounds with potential applications in health-related fields.

Phytochemical profiling of tomato aerial parts and axillary shoots revealed a complex composition of bioactive compounds, including a broad spectrum of phenolic acids and flavonols, with rutin emerging as the predominant metabolite. This compositional diversity contributes substantially to the antioxidant capacity of the plant extracts. Total phenolic content (TPC) and total flavonoid content (TFC), which serve as proxies for polyphenolic richness, were strongly correlated with DPPH inhibition activity, a commonly used indicator of antioxidant potential. These findings support the notion that phenolic compounds, particularly flavonoids such as rutin, play a key role in scavenging free radicals and modulating oxidative stress.

Using the Box–Behnken Design, we fine-tuned the extraction parameters and increased the recovery of antioxidant-active compounds. Extracts obtained at the optimum showed the highest TPC and TFC, with corresponding increases in antioxidant activity. Besides rutin, we identified caffeic acid, ferulic acid, esculetin, and spiraeoside—compounds widely reported for antioxidant effects. Their mechanisms include hydrogen donation, metal chelation, and inhibition of lipid peroxidation. Their co-occurrence in the optimized extracts suggests possible synergy and helps explain the stronger antioxidant outcomes. Overall, the direct link between phytochemical diversity and activity supports comprehensive profiling when evaluating plant by-products.

The results obtained, presented in Table 8, were compared with the existing literature data in order to highlight the variations in mineral concentrations based on the specific plant component and the extraction method used. The observed differences between the concentrations obtained may be attributed to the influence of the growing medium on the accumulation of minerals in the aerial parts of the plant. For example, tomato waste destined for valorization through anaerobic digestion presented different concentrations of nutrients (P, K, Ca, Mg, Mn, Fe, B, Cu, Zn), which were also influenced by the degree of shredding of the plant material [60]. In contrast, Almeida et al. [5] and Sanjuan-Delmás et al. [61] reported K as the predominant macronutrient, while Hurtado-Navarro et al. [62] identified notably higher concentrations of P and Fe specifically in tomato leaves. Another study [63] monitored the nutrient content of tomato leaves and stems following the application of sewage sludge biochar and compost, reporting substantial variations in macro- and micronutrient concentrations.

The detected macronutrients play an important role in carrying out metabolic functions, the detected micronutrients play a role in photosynthesis, stress resistance, and enzymatic activities, while the three beneficial elements help improve the structural integrity and stress tolerance of the tomato plant.

The results obtained in the current study regarding the elements P, K, Mg, Fe, Mn, Cu, Zn, and Na are in accordance with the studies mentioned above. In the extracts analyzed from aerial parts of tomato, K and Zn were found in significantly higher concentrations, thus highlighting the efficiency of MAE and UAE, as well as the possibility of these two extraction methods to improve the recovery process of a wide spectrum of elements. Moreover, K and Zn are involved in numerous biological processes at the human level. For example, K is involved in cellular functions. As well as in neuromuscular and cardiac activities, while Zn helps the good functionality of the immune system, as well as antioxidant and enzymatic activities. Therefore, the abundance of these two elements, K and Zn, in the extracts obtained from aerial parts of tomato, as well as those from axillary shoots, suggests their potential application in the pharmaceutical field as an important source of bioactive compounds with multiple health benefits.

The studied plant material is rich in secondary and primary metabolites, as well as macro- and microelements, which simultaneously contribute to improving health. For a nutraceutical exploitation, additional studies are needed to evaluate the bioavailability and therapeutic activity.

## 5. Conclusions

This study addresses a significant gap by performing the first comparative and optimized extraction of bioactive compounds—particularly rutin—from tomato aerial parts and axillary shoots using microwave-assisted (MAE) and ultrasound-assisted (UAE) techniques. Although tomato biomass is widely regarded as agricultural waste, its potential as a source of antioxidant compounds—especially in axillary shoots—has been underexplored. To our knowledge, no previous studies have systematically compared primary and secondary metabolite profiles between these plant components while optimizing extraction with a Box–Behnken design (BBD) combined with response surface methodology (RSM).

Using BBD–RSM, we optimized UAE and MAE to maximize TPC, TFC, DPPH, and rutin. Under the optimized settings, MAE applied to axillary shoots provided the highest rutin levels, while UAE favored the recovery of certain micronutrients and selected amino acids. Sequential (cascade) extraction further increased key constituents such as vitamin E and quinic acid. The close agreement between model predictions and experimental values supports the robustness of the optimization. Overall, matrix- and target-driven process selection enables efficient valorization of tomato biomass into phytochemically rich extracts suitable for functional applications.

The originality of this work lies not only in the targeted valorization of tomato plant waste but also in the development of green extraction protocols tailored to specific plant matrices. Building on these findings, future studies will evaluate the biological activities of extracts obtained under optimized conditions, including antimicrobial, antitumor (anticancer), and probiotic effects.

## Figures and Tables

**Table 1:** BBD experimental design.

Independent Variables	Unit	Level			Independent Variables	Unit	Level		
		−1	0	+1			−1	0	+1
		**MAE**					**UAE**		
A—Temperature	°C	70	98	125	A—Solvent concentration	% (*v*/*v*)	50	75	100
B—Solvent concentration	% (*v*/*v*)	50	75	100	B—Solvent to plant ratio	mL/g	20	30	40
C—Solvent to plant ratio	mL/g	20	30	40	C—Temperature	°C	40	55	70

**Table 2:** Response regression equation results.

Responses	Source	Sequential *p*-Value	R^2^	Adjusted R^2^	Predicted R^2^	Adeq. Precision	Model F-value	Mean	C.V. (%)
UAE of aerial parts of tomato waste (UAEap)									
TPC	Quadratic	<0.0001	0.9971	0.9933	0.9807	44.2159	265.25	6106.75	2.16
TFC			0.9972	0.9935	0.9547	52.0388	273.10	1672.24	1.92
DPPH			0.9924	0.9827	0.8845	35.3925	101.80	80.84	2.50
Rutin			0.9972	0.9937	0.9559	51.8733	280.41	4625.48	2.48
UAE of axillary shoots of tomato waste (UAEas)									
TPC	Quadratic	<0.0001	0.9982	0.9959	0.9800	68.6910	435.73	8304.62	2.27
TFC			0.9958	0.9904	0.9325	53.4503	183.63	2045.08	2.24
DPPH			0.9981	0.9958	0.9785	77.9718	418.84	88.12	0.16
Rutin			0.9916	0.9809	0.8663	34.2462	92.14	5674.95	3.14
MAE of aerial parts of tomato waste (MAEap)									
TPC	Quadratic	<0.0001	0.9867	0.9697	0.8072	20.3893	314.89	4702.95	4.70
TFC		<0.0001	0.9963	0.9915	0.9404	57.3852	207.84	1347.22	1.58
DPPH		0.0316	0.9898	0.9767	0.8590	30.9132	75.65	81.27	1.28
Rutin		<0.0001	0.9907	0.9787	0.8512	26.5370	82.71	4854.51	3.40
MAE of axillary shoots of tomato waste (MAEas)									
TPC	Quadratic	<0.0001	0.9877	0.9719	0.8078	23.3696	62.54	6730.53	3.73
TFC		0.0002	0.9928	0.9836	0.8855	42.2760	107.63	2196.13	3.37
DPPH		0.0037	0.9806	0.9556	0.9372	18.7854	39.26	89.65	0.45
Rutin		<0.0001	0.9914	0.9804	0.8631	35.7872	89.94	6380.13	2.74

**Table 3:** Optimum extraction conditions.

Extraction Method	Optimal Extraction Parameters			Desirability Function
	A	B	C	
UAEap	70%	36	56 °C	0.968
UAEas	65%	40	70 °C	0.867
MAEap	79 °C	39	78%	0.821
MAEas	125 °C	39	73%	0.858

**Table 4:** Experimental and maximum predicted response values at optimum extraction conditions.

Sample Response		UAEap	UAEas	MAEap	MAEas
TPC (mg GAE/kg)	Predicted	7684.54	12,267.00	5539.06	7851.48
	Experimental	7684.95 ± 2.23	12,269.40 ± 11.54	5539.89 ± 0.98	7853.15 ± 7.28
	*p*-value	0.8881	0.8737	0.8944	0.8609
TFC (mg QE/kg)	Predicted	2250.11	3057.93	1805.74	3503.00
	Experimental	2251.64 ± 1.87	3058.12 ± 2.15	1807.93 ± 0.65	3504.11 ± 2.78
	*p*-value	0.5520	0.9460	0.1001	0.7625
DPPH (%)	Predicted	86.995	87.778	85.174	89.133
	Experimental	87.023 ± 1.43	87.997 ± 0.61	85.853 ± 1.17	89.605 ± 0.86
	*p*-value	0.9880	0.7853	0.6651	0.6814
Rutin content (mg/kg)	Predicted	6646.03	8082.02	5745.95	8455.66
	Experimental	6647.15 ± 4.46	8083.24 ± 5.29	5746.07 ± 3.70	8455.85 ± 6.33
	*p*-value	0.8480	0.8602	0.9801	0.9816

**Table 5:** Cascade extraction parameters.

Sample Code	Plant Material	Extraction Time	Solvent to Plant Ratio	Solvent Concentration (%)
C1ap	Aerial parts	3 × 10 min pulses alternated with 2 × 15 min rest UAE + 1 h MAE	39	78
C1as	Axillary shoots		39	73
C2ap	Aerial parts	3 × 5 min pulses alternated with 2 × 7.5 min rest UAE + 40 min MAE	39	78
C2as	Axillary shoots		39	73

**Table 6:** LC-MS identified primary metabolites in the studied extracts.

Primary Metabolite	Extract (mg/g Dried Extract)							
	MAEap	UAEap	MAEas	UAEas	C1ap	C2ap	C1as	C2as
Amino Acids								
Serine	0.260 ± 0.009 ^b^	0.129 ± 0.004 ^c^	0.294 ± 0.037 ^b^	0.281 ± 0.004 ^b^	0.123 ± 0.001 ^c^	0.163 ± 0.009 ^c^	0.138 ± 0.001 ^c^	0.374 ± 0.004 ^a^
Glycine	0.045 ± 0.004 ^c^	0.041 ± 0.000 ^cd^	0.056 ± 0.003 ^b^	0.043 ± 0.002 ^cd^	0.034 ± 0.003 ^de^	0.026 ± 0.003 ^e^	0.082 ± 0.003 ^a^	0.056 ± 0.003 ^b^
Aspartic acid	3.547 ± 0.155 ^b^	5.065 ± 0.170 ^a^	1.402 ± 0.012 ^d^	3.164 ± 0.034 ^bc^	4.727 ± 0.072 ^a^	4.661 ± 0.599 ^a^	2.916 ± 0.084 ^bc^	2.512 ± 0.116 ^c^
Glutamine	0.146 ± 0.015 ^c^	nd	1.671 ± 0.047 ^b^	2.455 ± 0.115 ^a^	0.141 ± 0.006 ^c^	0.129 ± 0.017 ^c^	0.139 ± 0.006 ^c^	2.367 ± 0.065 ^a^
Sarcosine	0.580 ± 0.003 ^b^	0.878 ± 0.010 ^b^	2.517 ± 0.237 ^a^	0.882 ± 0.010 ^b^	0.784 ± 0.017 ^b^	2.454 ± 0.062 ^a^	0.827 ± 0.006 ^b^	2.332 ± 0.025 ^a^
Alanine	0.422 ± 0.030 ^b^	0.431 ± 0.006 ^b^	0.468 ± 0.057 ^ab^	0.536 ± 0.044 ^ab^	0.463 ± 0.018 ^ab^	0.444 ± 0.032 ^b^	0.501 ± 0.022 ^ab^	0.589 ± 0.007 ^a^
Threonine	0.804 ± 0.027 ^ab^	0.667 ± 0.020 ^d^	0.731 ± 0.010 ^c^	0.740± 0.008 ^bc^	0.655 ± 0.021 ^d^	0.659 ± 0.038 ^d^	0.653 ± 0.020 ^d^	0.827 ± 0.006 ^a^
Glutamic acid	2.289 ± 0.137 ^a^	1.460 ± 0.032 ^c^	0.666 ± 0.028 ^d^	1.806 ± 0.073 ^b^	1.496 ± 0.015 ^c^	2.105 ± 0.090 ^a^	0.831 ± 0.036 ^d^	1.532 ± 0.002 ^c^
Lysine	0.503 ± 0.012 ^c^	0.427 ± 0.000 ^d^	0.642 ± 0.021 ^b^	0.718 ± 0.007 ^a^	0.040 ± 0.004 ^f^	0.345 ± 0.000 ^e^	0.645 ± 0.013 ^b^	0.723 ± 0.009 ^a^
Proline	0.618 ± 0.010 ^c^	0.630 ± 0.007 ^c^	0.706± 0.005 ^ab^	0.675± 0.001 ^bc^	0.521 ± 0.023 ^d^	0.494 ± 0.013 ^d^	0.641 ± 0.028 ^c^	0.762 ± 0.000 ^a^
Histidine	0.787 ± 0.011 ^a^	0.692± 0.004 ^c^	0.542 ± 0.000 ^d^	0.767± 0.003 ^ab^	0.746 ± 0.025 ^ab^	0.739 ± 0.017 ^ab^	0.405 ± 0.018 ^e^	0.729 ± 0.020 ^bc^
2-Aminobutyric acid	0.200 ± 0.001 ^b^	nd	0.182 ± 0.001 ^c^	0.262 ± 0.001 ^a^	nd	nd	0.188 ± 0.003 ^c^	0.201 ± 0.003 ^b^
Arginine	0.064 ± 0.000 ^d^	0.064 ± 0.002 ^d^	0.892 ± 0.029 ^c^	1.252 ± 0.027 ^a^	0.054 ± 0.001 ^d^	0.050 ± 0.004 ^d^	0.892 ± 0.017 ^c^	1.167 ± 0.032 ^b^
Dimethylglycine	0.330 ± 0.011 ^c^	0.136 ± 0.009 ^d^	1.998 ± 0.000 ^a^	1.998 ± 0.071 ^a^	0.271 ± 0.008 ^c^	0.332 ± 0.012 ^c^	0.519 ± 0.010 ^b^	0.479 ± 0.029 ^b^
Valine	2.091 ± 0.007 ^d^	1.580 ± 0.025 ^e^	2.522 ± 0.101 ^c^	3.468 ± 0.052 ^b^	1.261 ± 0.024 ^f^	1.334 ± 0.000 ^ef^	1.385 ± 0.001 ^ef^	3.964 ± 0.235 ^a^
Isoleucine	1.160 ± 0.001 ^b^	1.032 ± 0.027 ^c^	1.208 ± 0.008 ^b^	1.310 ± 0.009 ^a^	0.979 ± 0.027 ^c^	1.011 ± 0.026 ^c^	1.132 ± 0.022 ^b^	1.208 ± 0.025 ^b^
Leucine	0.908 ± 0.032 ^c^	0.750 ± 0.025 ^d^	1.167 ± 0.025 ^b^	1.431 ± 0.043 ^a^	0.723 ± 0.019 ^d^	0.688 ± 0.001 ^d^	1.082 ± 0.027 ^b^	1.363 ± 0.034 ^a^
Phenylalanine	1.306 ± 0.030 ^d^	0.997 ± 0.002 ^e^	1.634 ± 0.022 ^c^	2.576 ± 0.022 ^a^	1.117 ± 0.044 ^e^	1.164 ± 0.031 ^de^	1.778 ± 0.022 ^c^	2.323 ± 0.121 ^b^
Tryptophan	4.449 ± 0.066 ^c^	3.517 ± 0.156 ^d^	3.674 ± 0.019 ^d^	5.395 ± 0.148 ^a^	3.402 ± 0.103 ^d^	4.772 ± 0.011 ^bc^	3.686 ± 0.130 ^d^	4.884 ± 0.066 ^b^
Vitamins								
Vitamin E	39.947 ± 0.101 ^e^	27.886 ± 0.580 ^f^	55.166 ± 1.213 ^c^	49.496 ± 1.940 ^d^	30.136 ± 1.260 ^f^	29.979 ± 0.029 ^f^	70.129 ± 0.437 ^a^	62.593 ± 2.097 ^b^
Organic Acids								
Quinic acid	29.899 ± 0.505 ^c^	nd	85.129 ± 3.458 ^b^	75.241 ± 0.152 ^b^	nd	nd	127.257 ± 0.000 ^a^	130.876 ± 10.466 ^a^
Malic acid	58.927 ± 3.209 ^a^	48.926 ± 0.662 ^c^	55.748 ± 1.297 ^ac^	54.828 ± 1.882 ^ac^	58.541 ± 0.756 ^a^	58.219 ± 1.184 ^ab^	52.746 ± 4.143 ^ac^	50.074 ± 1.707 ^bc^
Citric acid	5.875 ± 0.000 ^d^	14.069 ± 0.886 ^c^	11.865 ± 0.878 ^c^	36.053 ± 1.408 ^a^	nd	11.713 ± 0.002 ^c^	13.366 ± 0.542 ^c^	27.706 ± 0.863 ^b^
Plant hormones								
Indole-3-acetic acid	1.057 ± 0.058 ^d^	1.012 ± 0.005 ^d^	2.177 ± 0.087 ^c^	2.948 ± 0.074 ^a^	nd	nd	2.137 ± 0.077 ^c^	2.580 ± 0.102 ^b^
Nucleobases								
Cytosine	0.194 ± 0.003 ^a^	0.107 ± 0.003 ^e^	0.084 ± 0.002 ^f^	0.118 ± 0.004 ^d^	0.139 ± 0.003 ^c^	0.166 ± 0.006 ^b^	0.070 ± 0.000 ^g^	0.099 ± 0.004 ^e^
Alkaloids								
Histamine	0.342 ± 0.019 ^a^	0.163 ± 0.006 ^c^	0.174 ± 0.008 ^c^	0.307 ± 0.020 ^a^	0.326 ± 0.002 ^a^	0.250 ± 0.008 ^b^	nd	0.245 ± 0.008 ^b^
Quaternary ammonium compound								
Choline	1.078 ± 0.011 ^e^	0.486 ± 0.016 ^f^	1.256 ± 0.000 ^d^	1.647 ± 0.026 ^a^	0.331 ± 0.014 ^g^	nd	1.320 ± 0.000 ^c^	1.502 ± 0.027 ^b^

nd—not detected; the superscript letter within the same row indicates significant differences detected using ANOVA between studied samples (*p* < 0.05); data are presented as mean ± SD.

**Table 7:** LC-MS identified secondary metabolites in the studied extracts.

Secondary Metabolite	Extract (mg/g Dried Extract)							
	MAEap	UAEap	MAEas	UAEas	C1ap	C2ap	C1as	C2as
Lignans								
Coniferyl alcohol	0.111 ± 0.004 ^a^	nd	0.061 ± 0.002 ^c^	nd	0.032 ± 0.000 ^e^	0.032 ± 0.000 ^e^	0.083 ± 0.002 ^b^	0.043 ± 0.002 ^d^
Coumarins								
Esculin	0.006 ± 0.000 ^a^	0.003 ± 0.000 ^b^	nd	nd	0.004 ± 0.000 ^b^	0.004 ± 0.000 ^b^	nd	nd
Esculetin	0.035 ± 0.001 ^d^	nd	0.056 ± 0.000 ^c^	0.036 ± 0.001 ^d^	nd	nd	0.094 ± 0.001 ^a^	0.088 ± 0.001 ^b^
Flavones								
Baicalin	0.012 ± 0.001 ^a^	0.004 ± 0.000 ^d^	0.010 ± 0.001 ^b^	0.005 ± 0.000 ^c^	0.003 ± 0.000 ^de^	0.002 ± 0.000 ^e^	0.002 ± 0.000 ^e^	0.002 ± 0.000 ^e^
Anthocyanins								
Malvidin	18.069 ± 0.519 ^a^	15.195 ± 0.485 ^b^	6.528 ± 0.282 ^f^	9.503 ± 0.073 ^d^	12.676 ± 0.301 ^c^	18.946 ± 0.263 ^a^	7.498 ± 0.305 ^ef^	8.726 ± 0.341 ^de^
Flavonols								
Rutin	17.863 ± 0.046 ^b^	14.773 ± 0.026 ^c^	16.470 ± 0.182 ^bd^	17.429 ± 0.107 ^b^	14.849± 0.104 ^cd^	21.122 ± 0.766 ^a^	16.558 ± 0.504 ^b^	14.988 ± 0.599 ^c^
Isorquercitroside	0.094 ± 0.001 ^d^	0.039 ± 0.004 ^f^	0.143 ± 0.006 ^bc^	0.194 ± 0.004 ^a^	0.079 ± 0.000 ^e^	0.092 ± 0.004 ^d^	0.154 ± 0.001 ^b^	0.139 ± 0.004 ^c^
Kaempferol-7-O-glucoside	nd	nd	0.005 ± 0.000 ^b^	0.006 ± 0.000 ^a^	nd	nd	0.005 ± 0.000 ^b^	0.004 ± 0.000 ^c^
Kaempferol-7-O-neohesperidine	2.127 ± 0.058 ^c^	1.738 ± 0.037 ^c^	15.595 ± 0.504 ^a^	15.966 ± 0.603 ^a^	0.983 ± 0.692 ^c^	2.185 ± 0.044 ^c^	9.054 ± 6.400 ^b^	12.424 ± 0.230 ^ab^
Rhoifolin	0.004 ± 0.000 ^ab^	nd	0.004 ± 0.000 ^a^	0.004 ± 0.000 ^bc^	0.004 ± 0.000 ^c^	0.004 ± 0.000 ^bc^	0.004 ± 0.000 ^c^	0.004 ± 0.000 ^c^
Spiraeoside	nd	0.010 ± 0.001 ^e^	nd	0.026 ± 0.001 ^a^	0.008 ± 0.000 ^e^	0.014 ± 0.000 ^d^	0.024 ± 0.000 ^b^	0.022 ± 0.001 ^c^
Kaempferol-3-O-glucoside	nd	nd	0.035 ± 0.000 ^ab^	0.037 ± 0.001 ^a^	nd	nd	0.032 ± 0.001 ^b^	0.033 ± 0.001 ^ab^
Camelliaside A	0.227 ± 0.007 ^d^	0.139 ± 0.000 ^d^	1.748 ± 0.011 ^ab^	1.801 ± 0.067 ^a^	0.144 ± 0.001 ^d^	0.192 ± 0.003 ^d^	1.277 ± 0.068 ^c^	1.630 ± 0.041 ^b^
Isorhamnetin-3-O-rutinoside	0.056 ± 0.003 ^a^	0.044 ± 0.002 ^b^	0.046 ± 0.002 ^b^	0.042 ± 0.002 ^bc^	0.034 ± 0.001 ^d^	0.037 ± 0.000 ^cd^	0.036 ± 0.001 ^d^	0.043 ± 0.001 ^bc^
Phenolic Acids								
Protocatehuic acid	0.041 ± 0.000 ^ce^	0.064 ± 0.000 ^a^	0.040 ± 0.003 ^de^	0.037 ± 0.000 ^e^	0.046 ± 0.001 ^bc^	0.045 ± 0.001 ^bcd^	0.044 ± 0.003 ^cd^	0.050 ± 0.000 ^b^
Vanillic acid-4-O-glucoside	0.101 ± 0.001 ^a^	0.084 ± 0.001 ^b^	0.005 ± 0.000 ^c^	0.003 ± 0.000 ^c^	0.082 ± 0.001 ^b^	0.104 ± 0.003 ^a^	0.003 ± 0.000 ^c^	0.003 ± 0.000 ^c^
Gentisic acid	0.041 ± 0.001 ^cd^	0.059 ± 0.002 ^a^	0.041 ± 0.001 ^cd^	0.039 ± 0.000 ^d^	0.046 ± 0.001 ^bc^	0.051 ± 0.003 ^b^	0.042 ± 0.000 ^cd^	0.045 ± 0.001 ^c^
Neochlorogenic acid	1.206 ± 0.011 ^c^	0.644 ± 0.010 ^e^	1.116 ± 0.003 ^c^	1.863 ± 0.028 ^ab^	0.834 ± 0.031 ^d^	0.682 ± 0.001 ^e^	1.906 ± 0.055 ^a^	1.803 ± 0.031 ^b^
4-Hydroxybenzoic acid	0.018 ± 0.001 ^e^	0.012 ± 0.000 ^fg^	0.034 ± 0.002 ^c^	0.028 ± 0.001 ^d^	0.014 ± 0.000 ^f^	0.011 ± 0.001 ^g^	0.056 ± 0.001 ^a^	0.043 ± 0.000 ^b^
Chlorogenic acid	2.662 ± 0.100 ^d^	1.925 ± 0.065 ^e^	11.380 ± 0.352 ^c^	13.850 ± 0.250 ^a^	1.994 ± 0.066 ^de^	2.045 ± 0.094 ^de^	12.418 ± 0.313 ^b^	12.841 ± 0.398 ^b^
Cryptochlorogenic acid	0.368 ± 0.009 ^d^	0.239 ± 0.010 ^e^	1.746 ± 0.008 ^b^	1.039 ± 0.023 ^c^	0.264 ± 0.003 ^de^	0.244 ± 0.007 ^de^	3.292 ± 0.039 ^a^	1.739 ± 0.078 ^b^
Caffeic acid	0.022 ± 0.001 ^e^	nd	0.175 ± 0.001 ^c^	0.139 ± 0.005 ^d^	nd	nd	0.305 ± 0.005 ^a^	0.201 ± 0.000 ^b^
Ferulic acid	0.027 ± 0.000 ^a^	nd	0.023 ± 0.000 ^b^	nd	nd	nd	0.024 ± 0.001 ^b^	0.024 ± 0.000 ^b^
Salicyclic acid	0.044 ± 0.001 ^d^	0.018 ± 0.001 ^f^	0.086 ± 0.000 ^a^	0.076 ± 0.002 ^b^	0.023 ± 0.001 ^ef^	0.026 ± 0.000 ^e^	0.073 ± 0.002 ^b^	0.064 ± 0.003 ^c^
Caftaric acid	0.007 ± 0.000 ^c^	0.008 ± 0.000 ^c^	0.009 ± 0.000 ^b^	0.007 ± 0.000 ^c^	0.012 ± 0.000 ^a^	0.012 ± 0.001 ^a^	0.012 ± 0.000 ^a^	0.008 ± 0.000 ^c^

nd—not detected; the superscript letter within the same row indicates significant differences detected using ANOVA between studied samples (*p* < 0.05); data are presented as mean ± SD.

**Table 8:** ICP-OES analysis of plant extracts: Concentration of detected elements.

Element	MAEap	UAEap	MAEas	UAEas	C1ap	C2ap	C1as	C2as
Beneficial elements (mg/kg dried extract)								
Al	2.457 ± 0.187 ^cd^	2.147 ± 0.140 ^d^	5.257 ± 0.343 ^b^	2.826 ± 0.170 ^c^	6.479 ± 0.000 ^a^	2.265 ± 0.000 ^cd^	5.350 ± 0.111 ^b^	5.808 ± 0.000 ^b^
Li	8.038 ± 0.057 ^a^	8.007± 0.029 ^ab^	7.584 ± 0.110 ^c^	7.714 ± 0.013 ^bc^	7.972 ± 0.222 ^ab^	7.885 ± 0.082 ^ab^	7.709 ± 0.023 ^bc^	7.825 ± 0.130 ^ac^
Si	38.143 ± 1.445 ^c^	29.109 ± 1.313 ^d^	11.883 ± 0.636 ^ef^	4.747 ± 0.541 ^g^	54.544 ± 1.342 ^b^	60.238 ± 2.231 ^a^	14.183 ± 0.245 ^e^	7.436 ± 0.059 ^fg^
Micronutrients (mg/kg dried extract)								
B	46.019 ± 1.126 ^c^	56.290 ± 0.403 ^a^	22.344 ± 0.496 ^e^	12.261 ± 0.448 ^f^	51.877 ± 0.217 ^b^	52.007 ± 0.009 ^b^	27.060 ± 0.386 ^d^	23.616 ± 0.593 ^e^
Cu	15.635 ± 0.160 ^b^	22.204 ± 0.944 ^a^	9.193 ± 0.164 ^d^	21.494 ± 0.587 ^a^	22.293 ± 0.367 ^a^	20.640 ± 0.183 ^a^	12.670 ± 0.306 ^c^	13.402 ± 0.572 ^c^
Fe	0.996 ± 0.000 ^d^	1.501 ± 0.048 ^d^	9.318 ± 0.626 ^b^	3.733 ± 0.176 ^c^	5.113 ± 0.251 ^c^	1.537 ± 0.000 ^d^	12.993 ± 0.592 ^a^	9.577 ± 0.581 ^b^
Mn	4.644 ± 0.083 ^d^	17.904 ± 0.756 ^a^	3.009 ± 0.121 ^e^	18.672 ± 0.385 ^a^	5.286 ± 0.244 ^cd^	5.808 ± 0.171 ^cd^	6.000 ± 0.116 ^c^	7.354 ± 0.259 ^b^
Mo	7.733 ± 0.099 ^a^	7.908 ± 0.076 ^a^	7.719 ± 0.103 ^a^	7.776 ± 0.009 ^a^	7.734 ± 0.183 ^a^	7.844 ± 0.113 ^a^	7.742 ± 0.023 ^a^	7.858 ± 0.150 ^a^
Se	1.930 ± 0.149 ^d^	2.753 ± 0.019 ^ab^	2.900 ± 0.113 ^a^	2.387 ± 0.034 ^bc^	2.624 ± 0.027 ^ac^	2.292 ± 0.210 ^cd^	2.424 ± 0.126 ^bc^	2.885 ± 0.018 ^a^
Zn	35.989 ± 0.360 ^d^	42.466 ± 1.439 ^d^	67.645 ± 0.286 ^c^	105.393 ± 2.901 ^a^	43.968 ± 1.352 ^d^	45.292 ± 1.946 ^d^	84.504 ± 2.125 ^b^	94.880 ± 9.544 ^ab^
Macronutrients (g/kg dried extract)								
Ca	0.195 ± 0.003 ^c^	0.713 ± 0.021 ^a^	0.149 ± 0.009 ^d^	0.014 ± 0.003 ^f^	0.281 ± 0.010 ^b^	0.315 ± 0.003 ^b^	0.107 ± 0.001 ^de^	0.081 ± 0.005 ^e^
K	74.658 ± 0.402 ^ab^	77.979 ± 3.729 ^a^	58.024 ± 0.521 ^c^	70.623 ± 1.445 ^b^	71.150 ± 2.553 ^b^	73.681± 0.983 ^ab^	57.389 ± 0.355 ^c^	55.960 ± 1.245 ^c^
Mg	0.796 ± 0.012 ^e^	1.832 ± 0.064 ^b^	1.517 ± 0.049 ^c^	3.622 ± 0.072 ^a^	0.912 ± 0.045 ^de^	0.998 ± 0.042 ^d^	1.411 ± 0.017 ^c^	1.456 ± 0.042 ^c^
Na	1.131 ± 0.017 ^c^	1.320 ± 0.004 ^b^	0.580 ± 0.005 ^e^	0.670 ± 0.006 ^d^	1.302 ± 0.009 ^b^	1.360 ± 0.016 ^a^	0.583 ± 0.003 ^e^	0.543 ± 0.010 ^f^
P	2.065 ± 0.034 ^e^	3.491 ± 0.019 ^c^	4.150 ± 0.035 ^b^	7.369 ± 0.055 ^a^	2.248 ± 0.030 ^d^	2.408 ± 0.021 ^d^	4.138 ± 0.078 ^b^	4.166 ± 0.051 ^b^
S	0.981 ± 0.020 ^f^	1.691 ± 0.025 ^e^	1.874 ± 0.031 ^d^	3.386 ± 0.064 ^a^	1.035 ± 0.034 ^f^	1.072 ± 0.011 ^f^	2.611 ± 0.039 ^b^	2.320 ± 0.047 ^c^

The superscript letter within the same row indicates significant differences detected using ANOVA between studied samples (*p* < 0.05); data are presented as mean ± SD.

## Data Availability

All obtained datasets are contained within the article and Supplementary Materials.

## Supplementary Materials

The following supporting information can be downloaded at: https://www.mdpi.com/article/10.3390/antiox14091062/s1, Figure S1: Diagnostic plot of predicted versus experimental values for TPC, TFC and DPPH responses; Figure S2: 3D Response surface plots corresponding to the response regarding the TFC present in tomato waste; Figure S3: 3D Response surface plots corresponding to the response regarding the TPC present in tomato waste; Figure S4: 3D Response surface plots corresponding to the response regarding the DPPH present in tomato waste; Table S1: Experimental conditions for BBD, experimental and predicted values of responses; Table S2: Second-Order Polynomial Equations for the studied cases.
