# Supplementary material for: Box–Behnken Design Optimization of Green Extraction from Tomato Aerial Parts and Axillary Shoots for Enhanced Recovery of Rutin and Complementary Bioactive Compounds
Source: Antioxidants (Basel). 2025 Aug 29;14(9):1062. doi: 10.3390/antiox14091062 (PMC12466514; doi:10.3390/antiox14091062)
Supplement: Supplementary file 1 [file antioxidants-14-01062-s001.zip › antioxidants-3821667-supplementary.pdf]

antioxidants

Supplementary material

Box–Behnken Design optimization of green extraction from tomato aerial parts and axillary shoots for enhanced recovery of rutin and complementary bioactive compounds

Simona Marcu Spinu [1], Mihaela Dragoi Cudalbeanu [1,*], Nikola Major [2], Smiljana Goreta Ban [2], Igor Palčić [2], Alina Ortan [1], Petronela Mihaela Rosu [3,*] and Narcisa Elena Babeanu [4]

[1] Faculty of Land Reclamation and Environmental Engineering, University of Agronomic Sciences and Veterinary Medicine of Bucharest, 59 Marasti Boulevard, 011464 Bucharest, Romania; simona.spinu@fifim.ro (S.M.S.), mcudalbeanu@gmail.com (M.D.C.), alina.ortan@fifim.ro (A.O.)
[2] Institute of Agriculture and Tourism 52440 Poreč, Croatia; nikola@iptpo.hr (N.M.), smilja@iptpo.hr (S.G.B.), palcic@iptpo.hr (I.P.)
[3] Faculty of Veterinary Medicine, University of Agronomic Sciences and Veterinary Medicine of Bucharest, 59 Marasti Blvd, District 1, 011464 Bucharest, Romania; petronela.rosu@fmvb.usamv.ro (P.M.R.)
[4] Faculty of Biotechnologies, University of Agronomic Sciences and Veterinary Medicine of Bucharest, 59 Marasti Boulevard, 011464 Bucharest, Romania; narcisa.babeanu@biotehnologii.usamv.ro (N.E.B.)
* Correspondence: mcudalbeanu@gmail.com; petronela.rosu@fmvb.usamv.ro

Table S1. Experimental conditions for BBD, experimental and predicted values of responses.

UAE of aerial parts of tomato waste											
Run	Independent Factors			Response 1 (Y_1) TPC (mgGAE/kg dw)		Response 2 (Y_2) TFC (mgQE/kg dw)		Response 3 (Y_3) DPPH (%)		Response 4 (Y_4) Rutin (mg/kg)	
	A	B	C	Experimental	Predicted	Experimental	Predicted	Experimental	Predicted	Experimental	Predicted
1	0	0	0	7480.21	7475.57	2083.34	2083.15	89.21	89.53	6200.01	6200.77
2	0	-1	-1	6180.27	6110.64	1411.04	1396.73	82.70	82.72	3809.71	3736.66
3	-1	+1	0	7424.35	7421.28	2250.08	2233.31	66.57	64.67	6488.71	6501.17
4	+1	0	-1	3265.41	3331.97	952.54	950.08	85.11	83.19	1854.25	1939.76
5	0	-1	+1	5879.01	5845.62	1372.08	1334.32	86.81	86.66	3934.15	3868.87
6	0	0	0	7456.60	7475.57	2084.53	2083.15	89.87	89.53	6210.80	6200.77
7	0	+1	-1	6658.22	6691.61	1604.52	1642.28	78.39	78.54	4563.94	4629.22
8	0	+1	+1	6252.47	6322.10	1991.19	2005.50	90.28	90.26	5299.17	5372.22
9	-1	0	-1	7682.48	7652.16	1395.82	1374.82	43.37	45.12	4020.18	3942.44
10	0	0	0	7692.67	7475.57	2082.98	2083.15	90.16	89.53	6207.12	6200.77
11	-1	0	+1	6454.80	6388.24	1511.59	1514.05	54.01	55.93	4279.31	4193.80
12	0	0	0	7449.57	7475.57	2079.12	2083.15	88.62	89.53	6198.64	6200.77
13	-1	-1	0	5918.91	6018.86	1486.38	1521.69	60.11	58.33	3855.87	4006.66
14	+1	+1	0	3273.99	3174.04	1601.79	1566.48	91.35	93.13	3538.95	3388.16
15	0	0	0	7298.79	7475.57	2085.77	2083.15	89.80	89.53	6187.30	6200.77
16	+1	0	+1	3931.05	3961.37	1090.65	1111.65	89.79	88.04	2485.86	2563.60
17	+1	-1	0	3515.96	3519.03	1344.61	1361.38	98.14	100.00	3499.22	3486.76

UAE of axillary shoots of tomato waste											
Run	Independent Factors			Response 1 (Y_1) TPC (mgGAE/kg dw)		Response 2 (Y_2) TFC (mgQE/kg dw)		Response 3 (Y_3) DPPH (%)		Response 4 (Y_4) Rutin (mg/kg)	
	A	B	C	Experimental	Predicted	Experimental	Predicted	Experimental	Predicted	Experimental	Predicted
1	0	+1	+1	11083.00	11267.01	2964.22	3001.84	88.63	88.60	7715.77	7925.14
2	0	0	0	6023.92	6063.11	1886.56	1887.91	88.39	88.42	6196.60	6186.06
3	0	0	0	5963.34	6063.11	1887.18	1887.91	88.44	88.42	6188.30	6186.06
4	0	+1	-1	9067.36	9152.25	2431.38	2476.66	88.04	87.90	6600.00	6554.92

5	0	0	0	6296.54	6063.11	1889.83	1887.91	88.28	88.42	6194.75	6186.06
6	+1	0	-1	4623.38	4654.41	1139.37	1125.57	90.52	90.54	3155.53	3351.96
7	-1	+1	0	10036.2	9883.18	2508.21	2456.79	85.84	85.89	6066.07	6053.13
8	0	0	0	6094.6	6063.11	1888.74	1887.91	88.56	88.42	6180.90	6186.06
9	+1	0	+1	8440.16	8372.08	1781.37	1775.23	90.09	90.00	4781.64	4723.62
10	+1	-1	0	6087.37	6240.35	1260.31	1311.73	91.18	91.13	3231.76	3244.70
11	-1	0	+1	13783.8	13752.76	2337.27	2351.07	85.96	85.94	7029.70	6833.27
12	0	0	0	5937.13	6063.11	1887.26	1887.91	88.45	88.42	6169.75	6186.06
13	+1	+1	0	5686.55	5570.63	1671.43	1639.95	91.44	91.56	4360.13	4208.78
14	-1	0	-1	8417.36	8485.45	2195.24	2201.38	83.16	83.25	4898.45	4956.47
15	-1	-1	0	11023.6	11139.52	2115.04	2146.52	85.57	85.45	4963.14	5114.49
16	0	-1	-1	7921.56	7737.55	2320.55	2282.93	87.04	87.07	5558.93	5349.56
17	0	-1	+1	14692.6	14607.76	2602.36	2557.08	88.40	88.54	7182.71	7227.79

**MAE of aerial parts of tomato waste**											
**Run**	**Independent Factors**			**Response 1 (Y_1) TPC (mgGAE/kg dw)**		**Response 2 (Y_2) TFC (mgQE/kg dw)**		**Response 3 (Y_3) DPPH (%)**		**Response 4 (Y_4) Rutin (mg/kg)**	
	**A**	**B**	**C**	**Experimental**	**Predicted**	**Experimental**	**Predicted**	**Experimental**	**Predicted**	**Experimental**	**Predicted**
1	+1	+1	0	3923.29	3745.79	1025.91	1011.14	85.36	84.91	3634.68	3540.93
2	-1	0	-1	4089.23	3891.26	1302.21	1305.88	87.93	87.23	4097.94	4092.34
3	0	0	0	6252.88	6288.68	1463.99	1463.66	81.01	81.52	6194.76	6200.70
4	0	+1	-1	2846.66	2832.31	1061.03	1048.15	90.70	90.23	2963.12	2840.28
5	0	+1	+1	3628.36	3608.14	1494.93	1513.60	90.06	89.81	4259.87	4350.26
6	+1	-1	0	4672.93	4460.24	983.00	974.13	67.57	66.39	4443.60	4316.80
7	-1	+1	0	3304.97	3517.03	1170.22	1179.20	89.81	90.97	3177.42	3303.63
8	-1	-1	0	3539.16	3717.28	1258.47	1273.14	75.51	75.97	4747.34	4841.68
9	0	0	0	6191.88	6288.68	1464.07	1463.66	81.87	81.52	6204.09	6200.70
10	0	0	0	6391.88	6288.68	1463.10	1463.66	81.66	81.52	6217.62	6200.70
11	+1	0	+1	4126.31	4324.39	1237.37	1233.13	75.66	76.36	4086.00	4087.72
12	0	-1	+1	3201.54	3215.89	1215.22	1228.10	69.91	70.38	3984.10	4106.94
13	-1	0	+1	5445.79	5253.58	1939.37	1912.06	86.13	85.21	5685.89	5470.94

14	0	0	0	6384.88	6288.68	1462.78	1463.66	82.14	81.52	6188.56	6200.70
15	+1	0	-1	5600.06	5792.16	1489.86	1517.75	79.51	80.43	4969.16	5187.99
16	0	-1	-1	4128.4	4148.62	1406.87	1388.20	75.84	76.09	5474.05	5383.66
17	0	0	0	6221.88	6288.68	1464.34	1463.66	80.94	81.52	6198.48	6200.70

**MAE of axillary shoots of tomato waste**											
**Run**	**Independent Factors**			**Response 1 (Y_1) TPC (mgGAE/kg dw)**		**Response 2 (Y_2) TFC (mgQE/kg dw)**		**Response 3 (Y_3) DPPH (%)**		**Response 4 (Y_4) Rutin (mg/kg)**	
	**A**	**B**	**C**	**Experimental**	**Predicted**	**Experimental**	**Predicted**	**Experimental**	**Predicted**	**Experimental**	**Predicted**
1	+1	0	+1	7458.31	7347.75	3502.3	3588.86	89.26	89.28	8614.23	8649.86
2	+1	-1	0	8613.23	8395.59	3030.82	3001.60	86.27	86.40	6985.64	7154.89
3	0	0	0	8108.23	8068.23	2130.37	2132.14	90.08	90.48	6509.43	6511.45
4	-1	+1	0	5930.80	6141.93	1160.15	1189.03	91.61	91.48	4017.67	3854.12
5	0	+1	+1	4102.68	4067.08	1737.91	1663.04	92.07	92.16	4991.08	5103.39
6	0	0	0	8005.23	8068.23	2132.25	2132.14	90.73	90.48	6499.21	6511.45
7	+1	+1	0	5943.69	6090.50	2071.43	2061.09	91.49	91.38	5299.62	5149.64
8	0	0	0	8125.23	8068.23	2140.7	2132.14	90.49	90.48	6511.07	6511.45
9	0	-1	+1	4147.43	4469.77	3154.25	3097.93	86.95	86.79	8314.72	8113.51
10	0	+1	-1	4997.80	4675.46	1440.67	1496.99	91.78	91.94	3969.21	4170.43
11	0	0	0	8025.23	8068.23	2127.84	2132.14	89.93	90.48	6529.97	6511.45
12	-1	0	+1	6261.49	6085.31	1985.46	2030.08	89.44	89.48	6680.21	6733.49
13	-1	0	-1	6800.87	6906.22	2203.07	2118.89	89.48	89.46	6942.53	6908.52
14	-1	-1	0	5559.68	5419.38	2168.9	2179.57	86.30	86.41	7195.81	7340.09
15	0	0	0	8077.23	8068.23	2129.54	2132.14	91.16	90.48	6507.55	6511.45
16	0	-1	-1	5874.76	5910.36	1917.37	1992.24	87.35	87.26	6736.91	6624.60
17	+1	0	-1	8387.17	8568.56	2301.21	2254.20	89.59	89.55	6157.37	6102.47

Table S2. Second-Order Polynomial Equations for the studied cases

Responses	Equation
	**UAE of aerial parts of tomato waste**
**Response 1 (Y_1)** **TPC (mgGAE/kg dw)**	$TPC = 7475.57 - 1686.77\,A + 264.36\,B - 158.63\,C - 436.85\,AB + 473.33\,AC - 26.12\,BC - 1675.66\,A^2 - 766.60\,B^2 - 466.47\,C^2$
**Response 2 (Y_2)** **TFC (mgQE/kg dw)**	$TFC = 2083.15 - 206.78\,A + 229.18\,B + 75.20\,C - 126.63\,AB + 5.59\,AC + 106.41\,BC - 384.74\,A^2 - 27.69B^2 - 460.75\,C^2$
**Response 3 (Y_3)** **DPPH (%)**	$DPPH = 89.53 + 17.54\,A - 0.1458\,B + 3.91\,C - 3.31\,AB - 1.49\,AC + 1.94\,BC - 13.48\,A^2 + 2.99B^2 - 7.98\,C^2$
**Response 4 (Y_4)** **Rutin (mg/kg)**	$Rutin = 6200.77 - 908.22\,A + 598.98\,B + 218.80\,C - 648.28\,AB + 93.12\,AC + 152.70\,BC - 1548.46\,A^2 - 306.62\,B^2 - 1492.41\,C^2$
	**UAE of axillary shoots of tomato waste**
**Response 1 (Y_1)** **TPC (mgGAE/kg dw)**	$TPC = 6063.11 - 2302.93\,A - 481.51\,B + 2246.24\,C + 146.65\,AB - 387.41\,AC - 1188.86\,BC + 135.17\,A^2 + 2010.14\,B^2 + 2617.90\,C^2$
**Response 2 (Y_2)** **TFC (mgQE/kg dw)**	$TFC = 1887.91 - 412.91\,A + 159.62\,B + 199.84\,C + 4.49\,AB + 124.99\,AC + 62.76\,BC - 357.74\,A^2 + 358.57B^2 + 333.14\,C^2$
**Response 3 (Y_3)** **DPPH (%)**	$DPPH = 88.42 + 2.84\,A + 0.2207\,B + 0.5407\,C - 0.0034\,AB - 0.8074\,AC - 0.1916\,BC - 0.2558\,A^2 + 0.3384\,B^2 - 0.7358\,C^2$
**Response 4 (Y_4)** **Rutin (mg/kg)**	$Rutin = 6186.06 - 928.54\,A + 475.68\,B + 812.11\,C + 6.36\,AB - 126.29\,AC - 127.00\,BC - 1664.40\,A^2 + 133.62\,B^2 + 444.67\,C^2$
	**MAE of aerial parts of tomato waste**
**Response 1 (Y_1)** **TPC (mgGAE/kg dw)**	$TPC = 6284.44 + 242.93\,A - 228.68\,B - 26.36\,C - 128.55\,AB - 707.52\,AC + 427.14\,BC - 528.00\,A^2 - 1896.35\,B^2 - 941.09\,C^2$
**Response 2 (Y_2)** **TFC (mgQE/kg dw)**	$TFC = 1465.81 - 116.77\,A - 14.23\,B + 80.39\,C + 32.74\,AB - 222.70\,AC + 156.39\,BC - 80.43\,A^2 - 275.97\,B^2 + 106.83\,C^2$
**Response 3 (Y_3)** **DPPH (%)**	$DPPH = 81.60 - 3.91\,A + 8.38\,B - 1.52\,C + 0.8790\,AB - 0.5145\,AC + 1.32BC - 0.7119A^2 - 1.32\,B^2 + 1.42\,C^2$
**Response 4 (Y_4)** **Rutin (mg/kg)**	$Rutin = 6202.28 - 71.89\,A - 578.48\,B + 69.58\,C + 190.55\,AB - 619.72\,AC + 696.68\,BC - 831.82\,A^2 - 1369.70\,B^2 - 660.71\,C^2$

	MAE of axillary shoots of tomato waste
**Response 1 (Y_1)** **TPC (mgGAE/kg dw)**	$TPC = 8054.78 + 731.20\,A - 395.64\,B - 510.43\,C - 756.91\,AB - 99.98\,AC + 208.05\,BC + 458.40\,A^2 - 2001.34\,B^2 - 286.23\,C^2$
**Response 2 (Y_2)** **TFC (mgQE/kg dw)**	$TFC = 2124.37 + 423.52\,A - 482.76\,B + 311.46\,C + 12.51\,AB + 355.87\,AC - 234.91\,BC + 213.34\,A^2 - 229.89B^2 + 160.30C^2$
**Response 3 (Y_3)** **DPPH (%)**	$DPPH = 90.48 - 0.0275\,A + 2.51\,B - 0.0593\,C - 0.0247\,AB - 0.0719\,AC + 0.1725\,BC - 0.8285\,A^2 - 0.7328\,B^2 - 0.2077\,C^2$
**Response 4 (Y_4)** **Rutin (mg/kg)**	$Rutin = 6506.32 + 277.58\,A - 1372.80\,B + 593.09\,C + 370.18\,AB + 680.60\,AC - 138.99\,BC + 234.55\,A^2 - 866.18\,B^2 + 357.72\,C^2$

Figure S1. Diagnostic plot of predicted versus experimental values for TPC, TFC and DPPH responses

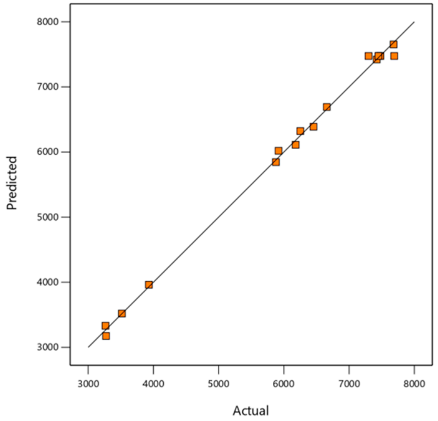

(a) TPC for UAEap

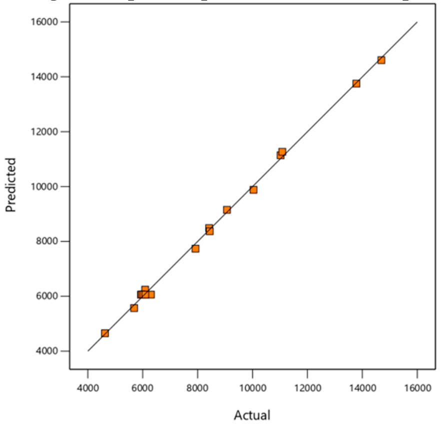

(b) TPC for UAEas

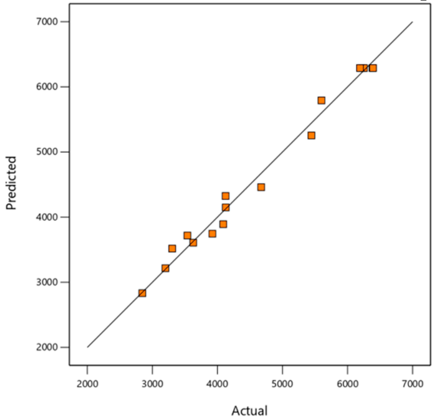

(c) TPC for MAEap

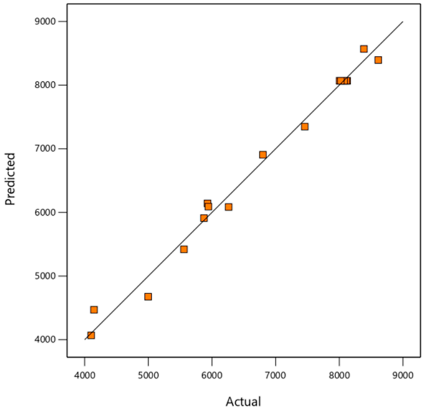

(d) TPC for MAEas

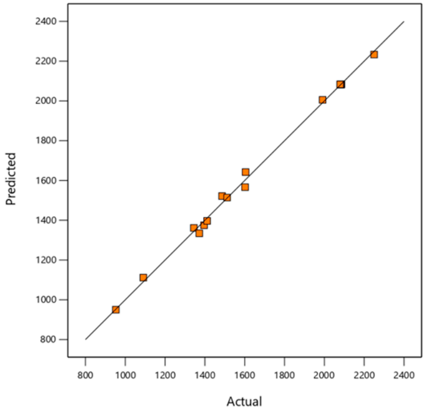

(e) TFC for UAEap

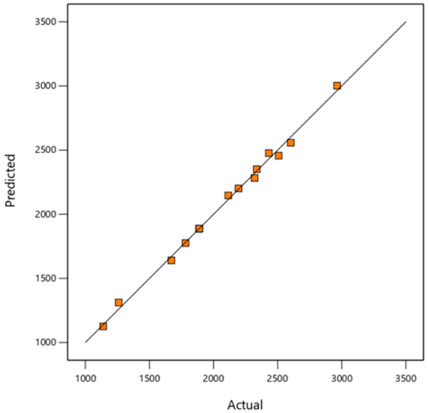

(f) TFC for UAEas

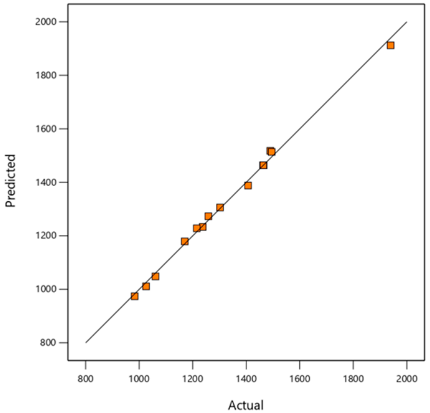

(g) TFC for MAEap

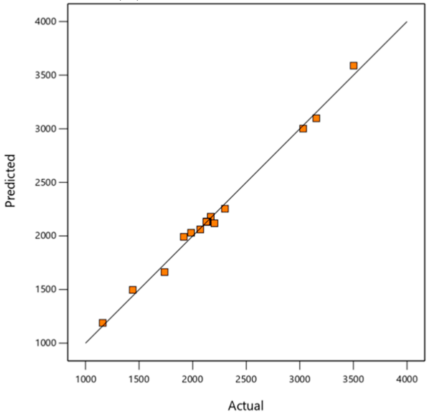

(h) TFC for MAEas

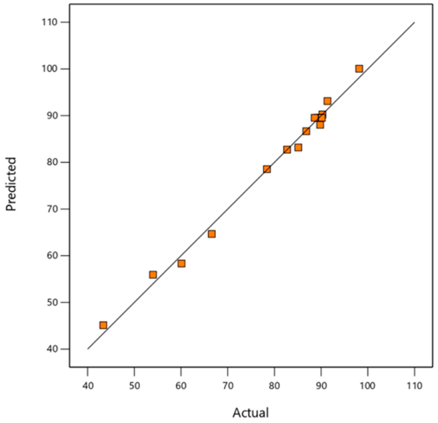

(i) DPPH for UAEap

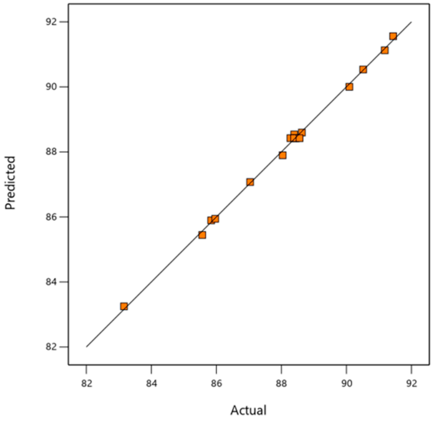

(j) DPPH for UAEas

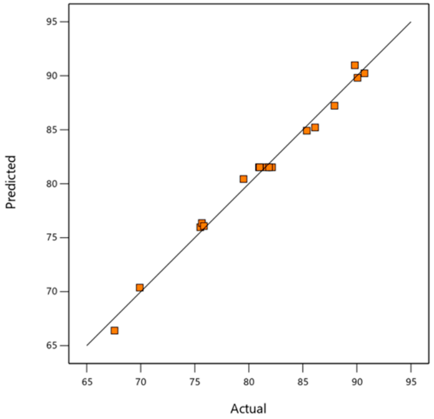

(k) DPPH for MAEap

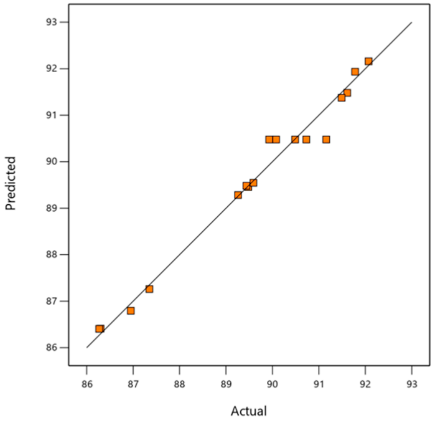

(l) DPPH for MAEas

Figure S2. 3D Response surface plots corresponding to the response regarding the TFC present in tomato waste

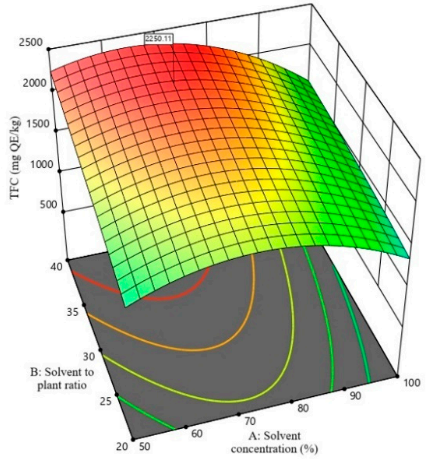

(a) Effect of solvent concentration and solvent to plant ratio on TFC for UAEap

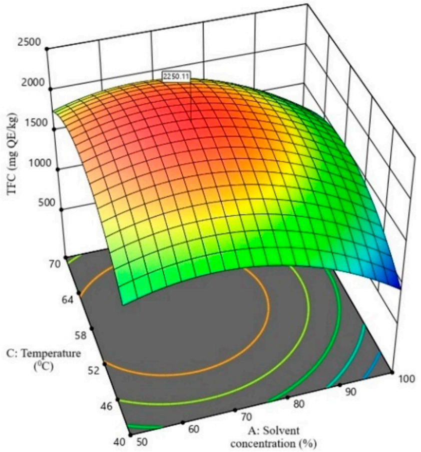

(b) Effect of solvent concentration and temperature on TFC for UAEap

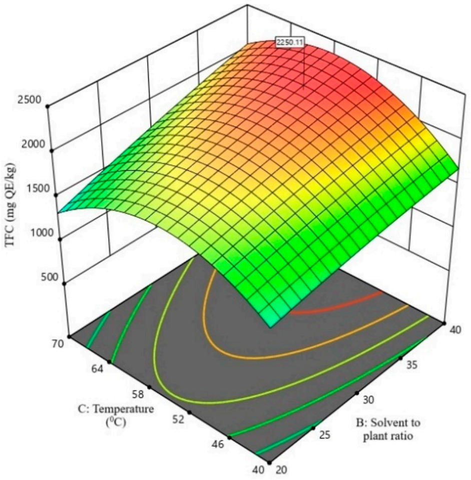

(c) Effect of solvent to plant ratio and temperature on TFC for UAEap

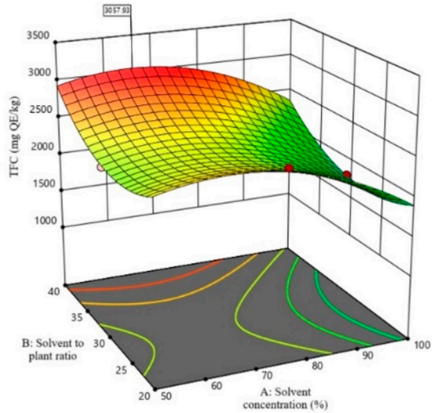

(d) Effect of solvent concentration and solvent to plant ratio on TFC for UAEas

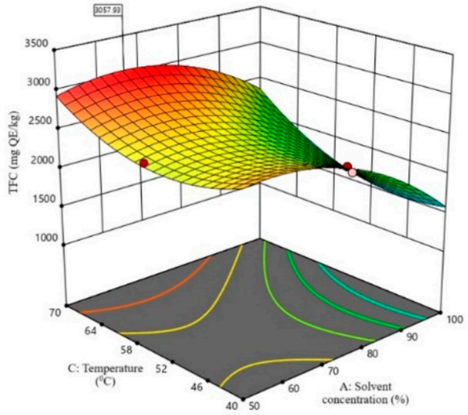

(e) Effect of solvent concentration and temperature on TFC for UAEas

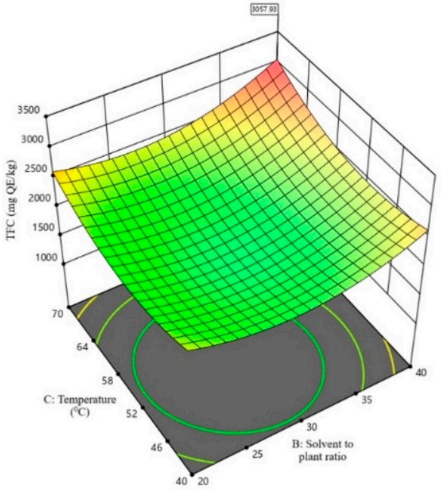

(f) Effect of solvent to plant ratio and temperature on TFC for UAEas

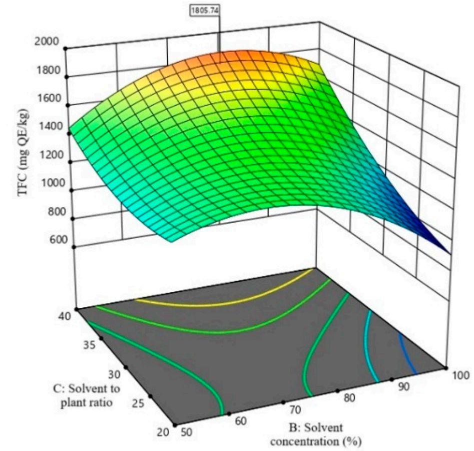

(g) Effect of solvent concentration and solvent to plant ratio on TFC for MAEap

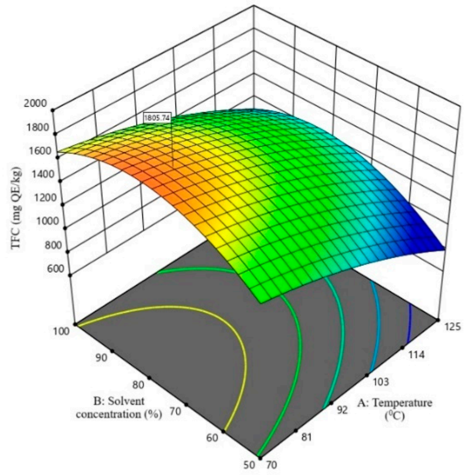

(h) Effect of solvent concentration and temperature on TFC for MAEap

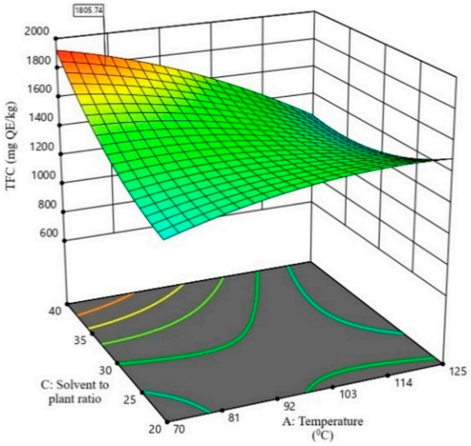

(i) Effect of solvent to plant ratio and temperature on TFC for MAEap

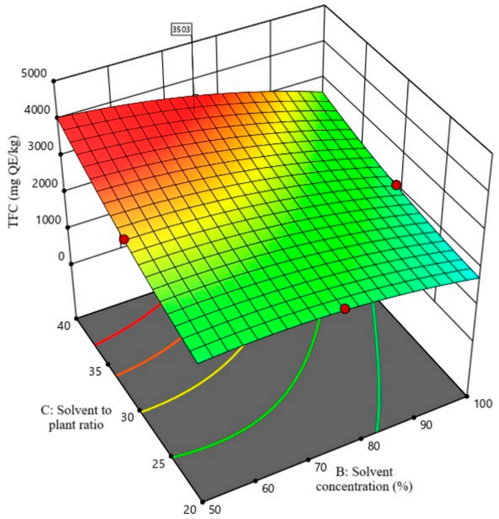

(j) Effect of solvent concentration and solvent to plant ratio on TFC for MAEas

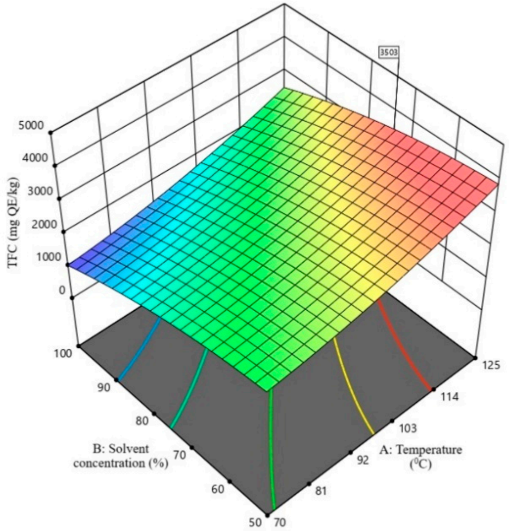

(k) Effect of solvent concentration and temperature on TFC for MAEas

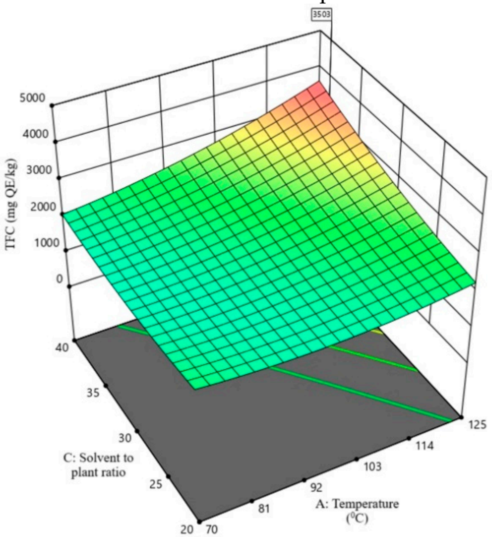

(l) Effect of solvent to plant ratio and temperature on TFC for MAEas

Figure S3. 3D Response surface plots corresponding to the response regarding the TPC present in tomato waste

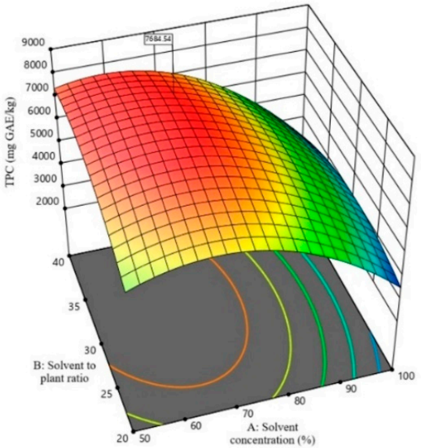

(a) Effect of solvent concentration and solvent to plant ratio on TPC for UAEap

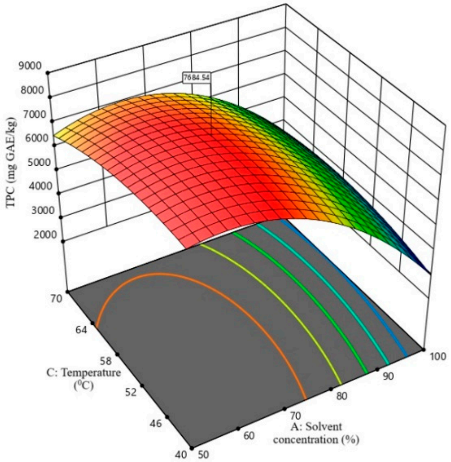

(b) Effect of solvent concentration and temperature on TPC for UAEap

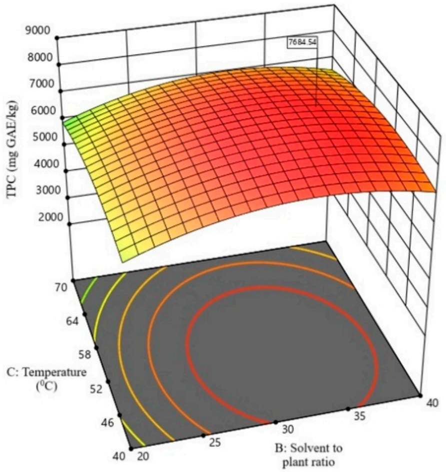

(c) Effect of solvent to plant ratio and temperature on TPC for UAEap

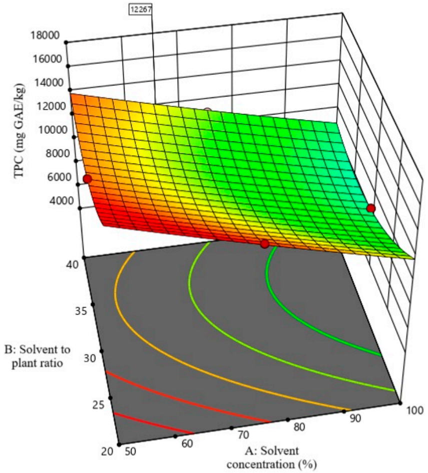

(d) Effect of solvent concentration and solvent to plant ratio on TPC for UAEas

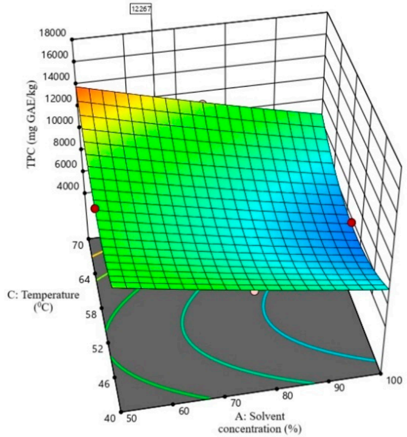

(e) Effect of solvent concentration and temperature on TPC for UAEas

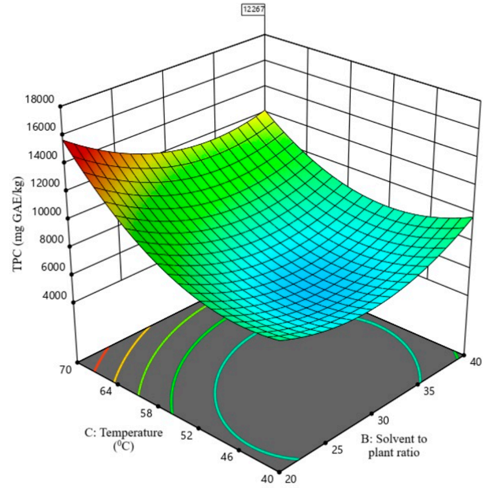

(f) Effect of solvent to plant ratio and temperature on TPC for UAEas

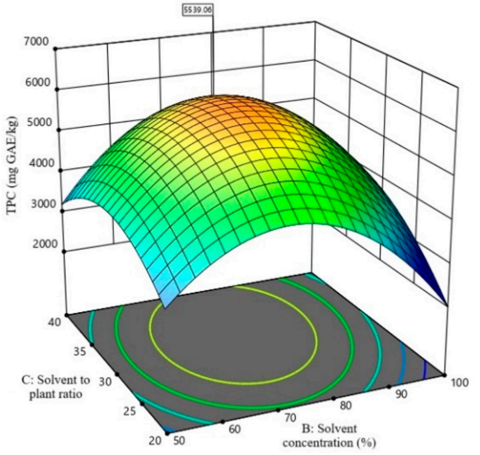

(g) Effect of solvent concentration and solvent to plant ratio on TPC for MAEap

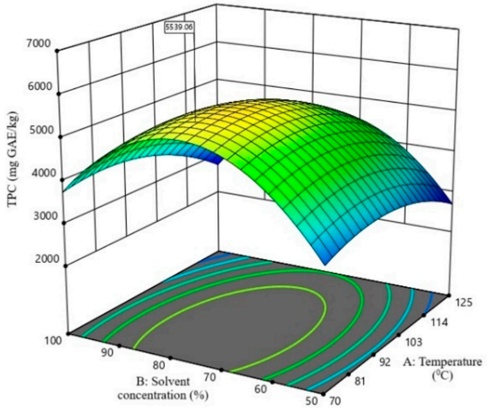

(h) Effect of solvent concentration and temperature on TPC for MAEap

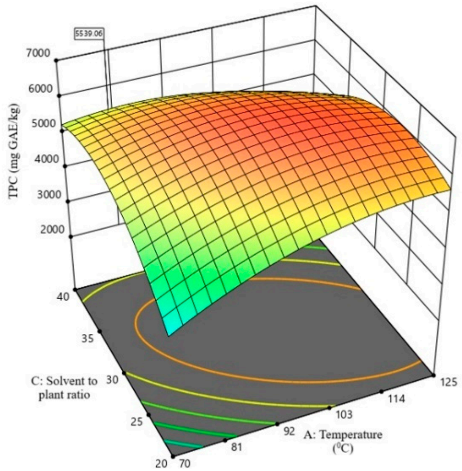

(i) Effect of solvent to plant ratio and temperature on TPC for MAEap

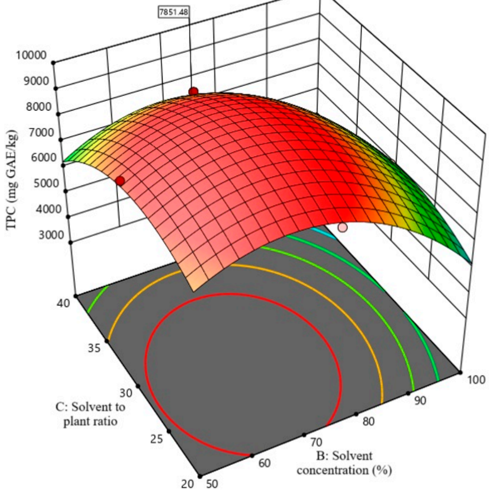

(j) Effect of solvent concentration and solvent to plant ratio on TPC for MAEas

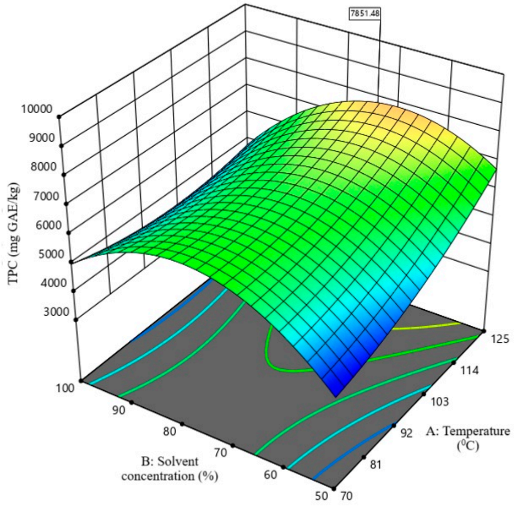

(k) Effect of solvent concentration and temperature on TPC for MAEas

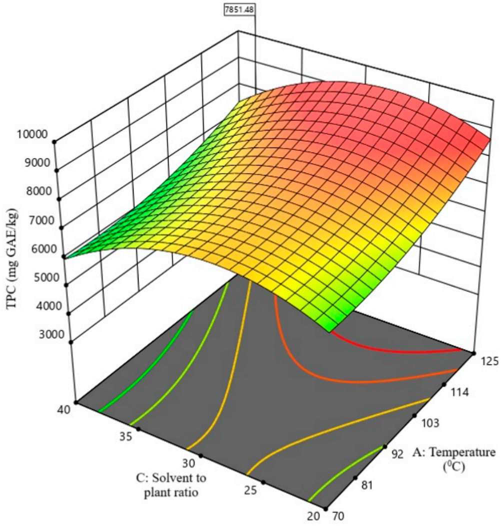

(l) Effect of solvent to plant ratio and temperature on TPC for MAEas

Figure S4. 3D Response surface plots corresponding to the response regarding the DPPH present in tomato waste

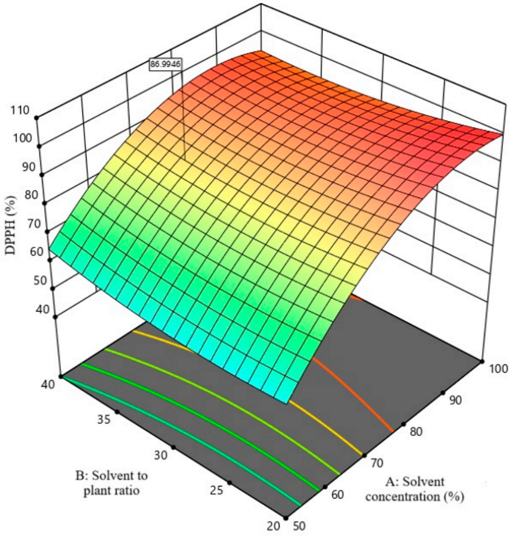

(a) Effect of solvent concentration and solvent to plant ratio on DPPH for UAEap

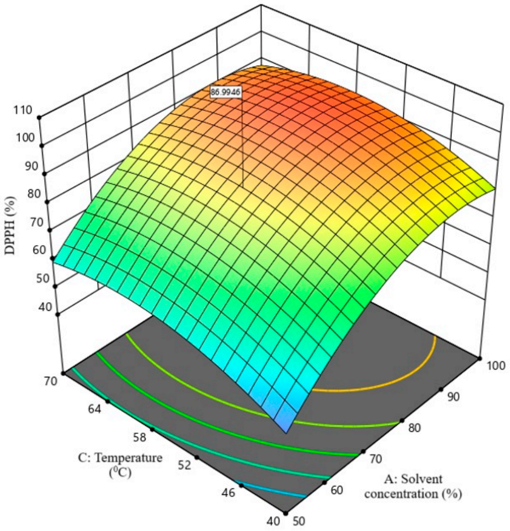

(b) Effect of solvent concentration and temperature on DPPH for UAEap

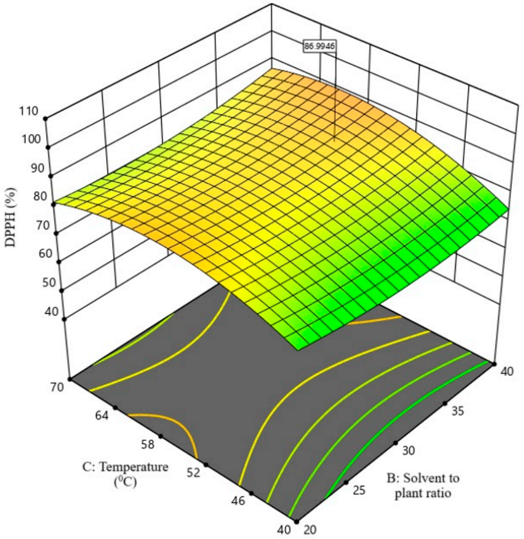

(c) Effect of solvent to plant ratio and temperature on DPPH for UAEap

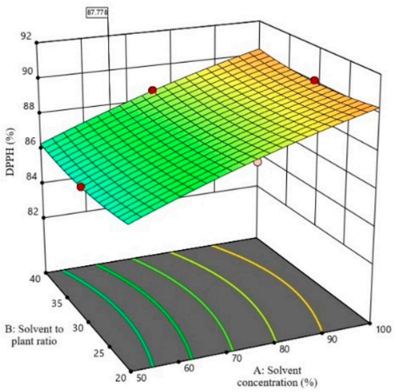

(d) Effect of solvent concentration and solvent to plant ratio on DPPH for UAEas

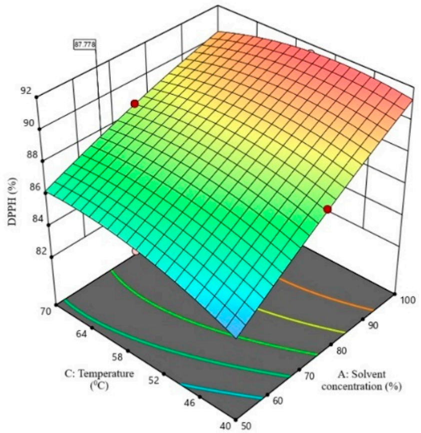

(e) Effect of solvent concentration and temperature on DPPH for UAEas

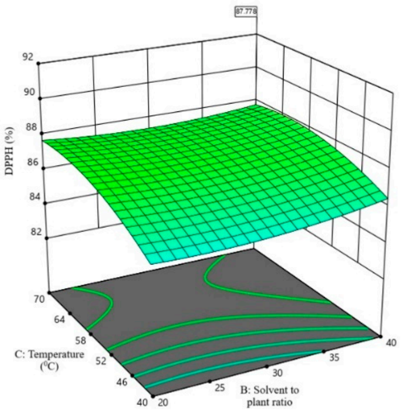

(f) Effect of solvent to plant ratio and temperature on DPPH for UAEas

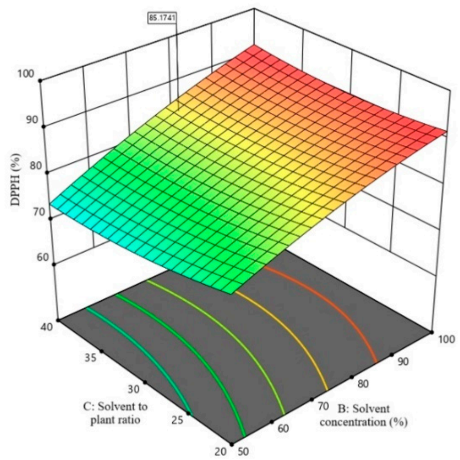

(g) Effect of solvent concentration and solvent to plant ratio on DPPH for MAEap

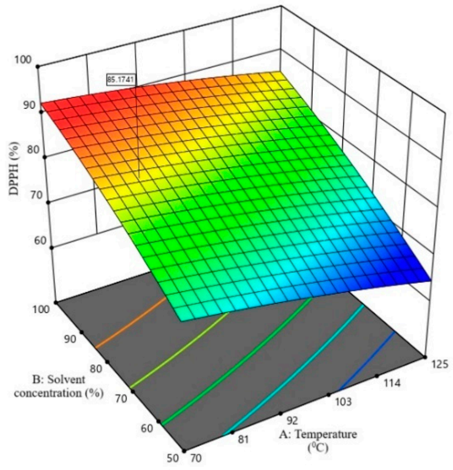

(h) Effect of solvent concentration and temperature on DPPH for MAEap

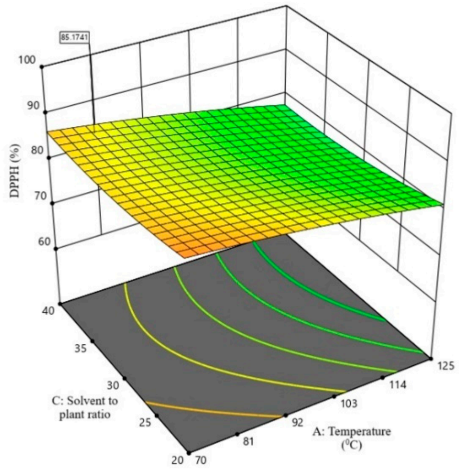

(i) Effect of solvent to plant ratio and temperature on DPPH for MAEap

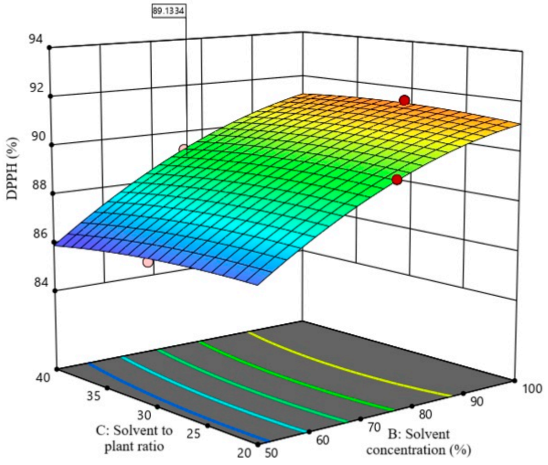

(j) Effect of solvent concentration and solvent to plant ratio on DPPH for MAEas

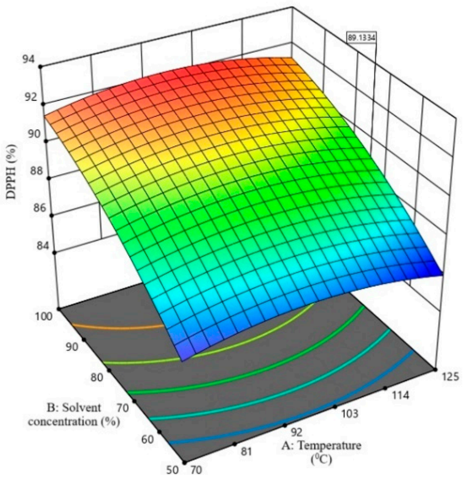

(k) Effect of solvent concentration and temperature on DPPH for MAEas

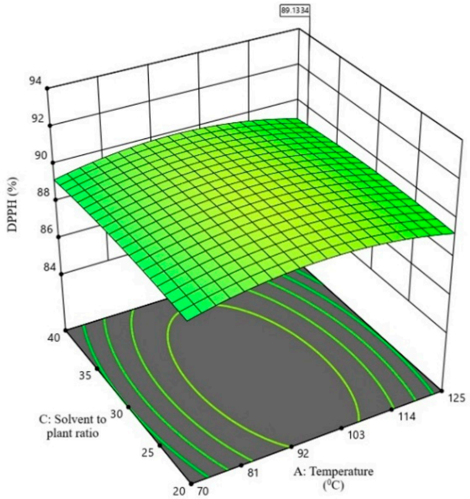

(l) Effect of solvent to plant ratio and temperature on DPPH for MAEas
